# Optogenetic modulation of guanine nucleotide exchange factors of Ras superfamily proteins directly controls cell shape and movement

**DOI:** 10.3389/fcell.2023.1195806

**Published:** 2023-07-10

**Authors:** Dhiman Sankar Pal, Yiyan Lin, Huiwang Zhan, Tatsat Banerjee, Jonathan Kuhn, Stephenie Providence, Peter N. Devreotes

**Affiliations:** ^1^ Department of Cell Biology and Center for Cell Dynamics, School of Medicine, Johns Hopkins University, Baltimore, MD, United States; ^2^ Department of Biological Chemistry, School of Medicine, Johns Hopkins University, Baltimore, MD, United States; ^3^ Department of Chemical and Biomolecular Engineering, Whiting School of Engineering, Johns Hopkins University, Baltimore, MD, United States; ^4^ Ingenuity Research Program, Baltimore Polytechnic Institute, Baltimore, MD, United States

**Keywords:** optogenetics, development, growth factor signaling, actin cytoskeleton, immunity, cancer metastasis, diabetes, neural networks

## Abstract

In this article, we provide detailed protocols on using optogenetic dimerizers to acutely perturb activities of guanine nucleotide exchange factors (GEFs) specific to Ras, Rac or Rho small GTPases of the migratory networks in various mammalian and amoeba cell lines. These GEFs are crucial components of signal transduction networks which link upstream G-protein coupled receptors to downstream cytoskeletal components and help cells migrate through their dynamic microenvironment. Conventional approaches to perturb and examine these signaling and cytoskeletal networks, such as gene knockout or overexpression, are protracted which allows networks to readjust through gene expression changes. Moreover, these tools lack spatial resolution to probe the effects of local network activations. To overcome these challenges, blue light-inducible cryptochrome- and LOV domain-based dimerization systems have been recently developed to control signaling or cytoskeletal events in a spatiotemporally precise manner. We illustrate that, within minutes of global membrane recruitment of full-length GEFs or their catalytic domains only, widespread increases or decreases in F-actin rich protrusions and cell size occur, depending on the particular node in the networks targeted. Additionally, we demonstrate localized GEF recruitment as a robust assay system to study local network activation-driven changes in polarity and directed migration. Altogether, these optical tools confirmed GEFs of Ras superfamily GTPases as regulators of cell shape, actin dynamics, and polarity. Furthermore, this optogenetic toolbox may be exploited in perturbing complex signaling interactions in varied physiological contexts including mammalian embryogenesis.

## Introduction

Directed cell migration is a highly orchestrated phenomenon fundamental to various physiological functions, including embryogenesis, wound healing, immune response, and cancer metastasis (Luster et al., 2005; Kruger et al., 2015; Devreotes et al., 2017; SenGupta et al., 2021). Dynamic, directed migratory events require precise spatiotemporal regulation of small GTPases, namely, Ras, Rac, and Rho. Guanine nucleotide exchange factors (GEFs) and GTPase-activating proteins (GAPs) perform this crucial function; GEFs catalyze the exchange of GDP to GTP to activate GTPases while GAPs accelerate GTP hydrolysis rate to turn them off. Since Ras GTPases transduce signals from chemoattractant-stimulated G-protein coupled receptors to downstream Rac and Rho GTPases to coordinate cytoskeletal activities, their respective GEFs and GAPs are vital network components of directed migration (Bos et al., 2007; Cherfils and Zeghouf, 2013; Artemenko et al., 2014; Devreotes et al., 2017; Pal et al., 2019; Gray et al., 2020).

Although GEFs have been implicated in migration through knockout, knockdown, or overexpression studies, it has been difficult to assign specific roles for them (Insall et al., 1996; Uhlenbrock et al., 2004; Francis et al., 2006; Nalbant et al., 2009; Pakes et al., 2012; Suire et al., 2012; Ding et al., 2018). This is largely because phenotypes observed from conventional genetic and biochemical assays require multiple days, or even months, to develop allowing sufficient time for signaling and cytoskeletal networks to re-adjust through differential gene expression or protein rearrangement (Kok et al., 2015; Rossi et al., 2015; Stainier et al., 2015; Morgens et al., 2016; El-Brolosy and Stainier, 2017; Housden et al., 2017; El-Brolosy et al., 2019). Moreover, these tools lack the spatial resolution to locally activate GEFs and examine their effects on confined actin organization and directed migration.

**FIGURE 1:**
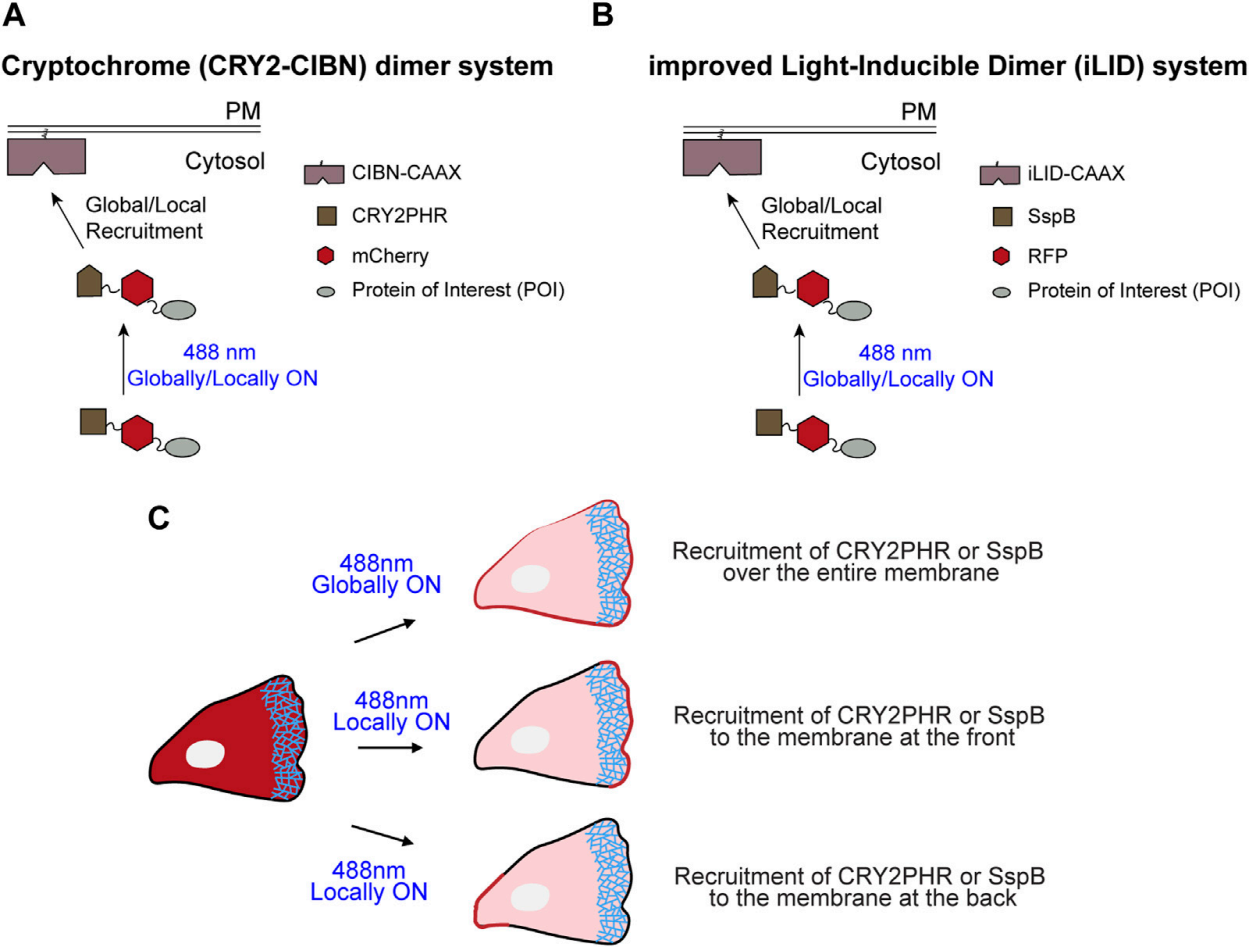
Schematic representation of cryptochrome- and improved light-inducible dimer (iLID)-based optogenetics. **(A)** Schematic illustrating recruitment of cytosolic CRY2PHR-mCherry fused with a protein of interest to the plasma membrane anchor, CIBN-CAAX, by global or local illumination with 488 nm laser. **(B)** Schematic demonstrating recruitment of cytosolic SspB-RFP fused with a protein of interest to the membrane anchor, iLID-CAAX, by global or local illumination with 488 nm light. **(C)** Depending on the region of the plasma membrane where 488 nm light was applied, CRY2PHR or SspB was recruited either all over the cell boundary (global recruitment), or specifically to the front or back of the cell (local recruitment).

In recent times, light-induced dimerization systems enabled local and reversible perturbation of upstream G-protein coupled receptor and Ras signaling, or the activities of downstream cytoskeletal components, such as Rho, Rac, or Cdc42, and steered migration. For acutely modulating the activities of these small GTPases, the catalytic domain of their respective GEFs was optically recruited to the plasma membrane (Yoo et al., 2010; O'Neill et al., 2018; Wu et al., 2009; O'Neill et al., 2016; Wu et al., 2011; Pal et al., 2023; Kato et al., 2014; de Beco et al., 2018; Bell et al., 2021; Valon et al., 2015; O'Neill and Gautam, 2014; Karunarathne et al., 2013; Krueger et al., 2019; Zhou et al., 2012; Wang et al., 2010). In this article, we demonstrate how to use these cryptochrome- and light-oxygen-voltage-sensing (LOV) domain-based optical tools to acutely clamp activities of Ras, Rac, and Rho GEFs on the cell membrane of different cell types (Figure 1). Our optical perturbations have brought forth a multitude of cytoskeletal and migratory responses which would have been challenging with previous methods evaluating GEF function.

## Materials and equipment

### Recombinant DNA used

#### HL-60 cells

eGFP-deleted pLJM1 lentiviral plasmid (Addgene #19319) expressing CIBN-CAAX (CIBN-CAAX/pLJM1; Addgene #201749) or LifeAct-miRFP703 (LifeAct-miRFP703/pLJM1; Addgene #201750) (Sancak et al., 2008; Idevall-Hagren et al., 2012; Shcherbakova et al., 2016; Pal et al., 2023).

PiggyBac™ transposon system consisting of a) pPB-bsr2 transposon plasmid expressing CRY2PHR-mCherry-RasGRP4 (Addgene #201754), and b) pCMV-hyPBase transposase expression plasmid (system obtained from Sean Collins Lab, UC Davis) (Yusa et al., 2009; Bell et al., 2021; Hadjitheodorou et al., 2021; Pal et al., 2023).

#### Dictyostelium

pDM358 plasmid (DictyBase #534) expressing cAR1-CIBN or CAAX-deleted Venus-iLID fused to N150 gene fragment (N150-Venus-iLID; Addgene #201763) (Gaudet et al., 2011; Banerjee et al., 2022; Pal et al., 2023).

Doxycycline-inducible pDM335 plasmid (DictyBase #523) expressing tgRFPt-SspB R73Q (Addgene #60416) (Veltman et al., 2009; Gaudet et al., 2011; Guntas et al., 2015).

pCV5 plasmid (DictyBase #23) expressing CRY2PHR-mCherry (Addgene #26866), or tgRFPt-SspB R73Q fused to the N-terminal of the catalytic domain of RacGEF1 (gift from Richard Firtel lab, UCSD) or CAAX-deleted KRas4B G12V (Addgene #9052) (Park et al., 2004; Gaudet et al., 2011; Miao et al., 2019; Banerjee et al., 2022).

#### MCF-10CA1h cells

pStargazin-GFP-LOVpep (Addgene #80406) (Wagner and Glotzer, 2016).

p2XPDZ-mCherry-LARG (DH) (Addgene #80407) (Wagner and Glotzer, 2016).

pLifeact-7-iRFP670 (Addgene #103032) (Padilla-Rodriguez et al., 2018).

#### RAW 264.7 cells

pCIBN-CAAX (Addgene #79574) (Idevall-Hagren et al., 2012).

pCRY2PHR (W349R)-mCherry (Addgene #75370) (Taslimi et al., 2016).

pCRY2low-tdTomato (Addgene #104067) (Duan et al., 2017).

pLL7.0-Venus-iLID-CAAX (Addgene #60411) (Guntas et al., 2015).

pLL7.0-tgRFPt-SspB R73Q (Addgene #60416) (Guntas et al., 2015).

### Cell culture reagents

For more details, see Supplementary Materials and Methods.

### *Dictyostelium* development/neutrophil differentiation system and reagents

For more details, see Supplementary Materials and Methods.

### Lentiviral transduction reagents

Packaging plasmids: pMDLg/pRRE (Addgene #12251), pMD2. G (Addgene #12259), and pRSV-Rev (Addgene #12253) (Kato et al., 2014; Dull et al., 1998)

HEK293T cell line (obtained from ATCC).

Lipofectamine 3000 Transfection Reagent (Invitrogen #L3000008).

Opti-MEM reduced serum medium (Gibco #31985-062).

10 cm cell culture dish.

6-well plate (Greiner Bio-One #657160).

Polybrene (Sigma #TR1003).

48-well cell culture plate (Sarstedt #83.3923).

DMEM (Gibco #10569-010) supplemented with 10% FBS and 1% penicillin-streptomycin.

### Transfection system and reagents

Neon™ transfection kit (Invitrogen #MPK10025B).

Neon™ electroporation system (Invitrogen #MPK5000).

Gene Pulser Electroporation Cuvettes, 0.1 cm gap (BIO-RAD #1652089).

Gene Pulser Xcell Electroporation System (BIO-RAD #1652660).

0.1 cm-gap cuvette (BIO-RAD, 1652089).

Lipofectamine 3000 Transfection Reagent.

10 cm cell culture dish.

48-well cell culture plate.

Opti-MEM reduced serum medium.

Amaxa cell line kit V (Lonza; #VACA-1003).

Amaxa Nucleofector II device (Amaxa Biosystems).

### Imaging system and reagents

Zeiss LSM800 GaAsP single-point, laser scanning confocal microscope with a wide-field camera.

Zeiss LSM780-FCS single-point, laser scanning confocal microscope (Zeiss Axio Observer with 780-Quasar confocal module).

8-well chamber (Lab-Tek, #155409 PK).

200 μg/mL fibronectin (Sigma #F4759).

MCF-10CA1h imaging medium: phenol red-free DMEM/F-12 (Gibco #21041-025).

RAW 264.7 imaging medium: phenol red-free HBSS (Gibco # 14025092) supplemented with 1 g/L glucose.

### Cell sorting system and reagents

BD FACSAria IIu cell sorter (Beckton Dickinson).

SH800S cell sorter (Sony).

40 µm nylon cell strainer (Corning #431750).

5 mL polystyrene round-bottom tube (Corning #352235).

100 μm cell sorting chip (Sony #LE-C3210).

Sorting buffer (1x PBS, Ca^2+^/Mg^2+^ free; 0.9% FBS; 2% penicillin-streptomycin).

Collection medium (RPMI medium 1640 supplemented with 20% FBS and 2% penicillin-streptomycin).

### Immunoblotting system

For more details, see Supplementary Materials and Methods.

## Methods

### Plasmid construction

For lentiviral constructs, CIBN-CAAX or LifeAct-miRFP703 ORF was cloned in NheI/EcoRI sites of pLJM1-eGFP plasmid, in place of the eGFP gene (Sancak et al., 2008; Idevall-Hagren et al., 2012; Shcherbakova et al., 2016; Pal et al., 2023). For transposon system, CRY2PHR-mCherry gene was first sub-cloned into the pPB-bsr2 transposon plasmid in XhoI/NotI sites. Next, at the C-terminal of CRY2PHR-mCherry gene in the transposon plasmid, we introduced full-length RasGRP4 gene at BspEI/SalI sites (Yusa et al., 2009; Kennedy et al., 2010; Bell et al., 2021; Pal et al., 2023).

For *Dictyostelium* constructs, N150-Venus-iLID gene was subcloned in pDM358 plasmid using AgeI/BamHI restriction digestion. The tgRFPt-SspB R73Q gene was subcloned in pCV5 or pDM335 plasmid using AgeI/BamHI or BglII restriction digestion (Guntas et al., 2015; Pal et al., 2023). Next, at the C-terminal of tgRFPt-SspB R73Q gene in pCV5, RacGEF (catalytic domain of RacGEF1) or KRas4B G12V ΔCAAX was PCR amplified and introduced via NheI/NotI digestion (Park et al., 2004; Miao et al., 2019). cAR1-CIBN was cloned into BglII/SpeI sites of pDM358 plasmid whereas CRY2PHR-mCherry gene was sub-cloned into XbaI/NheI in pCV5 plasmid (Banerjee et al., 2022).

All constructs were sequenced and verified at the JHMI Synthesis and Sequencing Facility.

### Cell culture

For more details, see Supplementary Materials and Methods.

### Development and differentiation of *dictyostelium* and neutrophils

For more details, see Supplementary Materials and Methods.

### Stable cell line construction

For stable protein expression in HL-60 cells, a combination of lentiviral and transposon approaches was used sequentially (Bell et al., 2021; Hadjitheodorou et al., 2021; Li et al., 2021; Pal et al., 2023). The procedure involved three steps. In step 1, virus was made using HEK293T cells at ∼80% confluency. For each transfection, 2 µg pMDLg/pRRE, 4.64 µg pRSV-Rev, 3.32 µg pMD2. G, and 10 µg CIBN-CAAX/pLJM1 constructs were mixed with Lipofectamine 3000 transfection reagent as per manufacturer’s instructions (Dull et al., 1998). After 96 h, virus-rich culture medium was collected at 3000 rpm for 20 min at 4°C. Next, viral supernatant was added to 4 × 10^6^ HL-60 cells, seeded at a density of 0.25 × 10^6^ cells/mL, in a 6-well plate. 10 μg/mL polybrene was added to this cell-virus mixture. Post 24 hour-incubation, virus was aspirated, and infected cells were added to a mixture of conditioned and fresh (mixed) medium. To select CIBN-CAAX-expressors, 1 μg/mL puromycin was added to infected cells after a day of recovery. Infected cells were kept in presence of puromycin for 5 days till only resistant cells were alive. Next, cells were harvested and transferred to a 48-well cell culture plate without puromycin for ∼3 weeks till resistant cells grew to confluency. These CIBN-CAAX expressing cells were next infected with LifeAct-miRFP703-expressing virus as explained above. To select LifeAct-miRFP703 expressors, cells were sorted on the fifth day post-infection, and grown to confluency. Finally, cells expressing CIBN-CAAX and LifeAct-miRFP703 were maintained in puromycin.

Next, recruitable RasGRP4 was introduced in CIBN-CAAX and LifeAct-miRFP703 dual expressing HL-60 cells using PiggyBac transposon system. 5 μg CRY2PHR-mCherry-RasGRP4/pPB plasmid was co-electroporated with an equal amount of transposase expression plasmid (pCMV-hyPBase) into 2 × 10^6^ cells using Neon transfection kit. DNA-cell mixture was resuspended in buffer “R” before single-pulse electroporation was carried out in a 100 µL pipette at 1350 V for 35 m sec using Neon electroporation system. Cells were resuspended in mixed culture medium in a 6-well plate and selected in presence of 10 μg/mL blasticidine S, as described for puromycin. Finally, these triple expressors were cultured throughout in puromycin and blasticidine S.

*Dictyostelium* stable cell lines were generated by electroporation (Li et al., 2021; Banerjee et al., 2022; Banerjee et al., 2023; Pal et al., 2023). Briefly, 1 × 10^7^ cells were harvested, washed twice, and resuspended in 100 μL ice-cold H-50 buffer. Next, 2 μg iLID (tgRFPt-SspB R73Q/pDM335, tgRFPt-SspB R73Q-RacGEF/pCV5 or tgRFPt-SSPB R73Q-KRas4B G12V/pCV5 and N150-Venus-iLID/pDM358) or cryptochrome (CRY2PHR-mCherry/pCV5 and cAR1-CIBN/pDM358) constructs were mixed with the cell suspension, and moved to an ice-cold 0.1 cm-gap cuvette. Electroporation was done with two pulses at 0.85 kV/25 μF at 5 s interval. Cuvettes were then incubated on ice for 10 min. Next, cells were transferred to a 10 cm culture dish containing 10 mL HL5 culture medium supplemented with heat-killed bacteria. On the next day, 10–20 μg/mL G418 sulphate and 30–40 μg/mL hygromycin B were added to cells and selected over 3–4 weeks. To induce protein expression, doxycycline (50 μg/mL) was added to the selected cells, 8–12 h prior to imaging. Cells were placed in an 8-well cover slip chamber and allowed to adhere for 20 min. HL5 medium was aspirated and 300 μL DB was added to cells. After a further 30 min incubation in the dark, cells were taken for imaging.

### Transient transfection

RAW 264.7 cells were transfected by nucleofection using Amaxa cell line kit V (Banerjee et al., 2022; Pal et al., 2023). Briefly, 3 × 10^6^ cells were harvested and resuspended in 100 µL Nucleofector Solution V containing 1.8 µg cryptochrome [0.7 µg pCIBN-CAAX and 1.1 µg pCRY2PHR (W349R)-mCherry or pCRY2 (R489E, A491D)-tdTomato] or iLID (0.9 µg pLL7.0-tgRFPt-SspB R73Q and pLL7.0-Venus-iLID-CAAX each) construct (Guntas et al., 2015; Taslimi et al., 2016; Duan et al., 2017). DNA and cell were mixed gently, transferred to a Lonza cuvette, and electroporated with Amaxa Nucleofector II device using the pre-set program “D-32”. Cell-DNA mix was transferred to 500 µL pre-warmed culture medium after a single pulse, and incubated for 10 min at 37°C and 5% CO_2_. Next, 2 × 10^5^ cells were added to an 8-well chambered cover glass and incubated for 1 h 500 μL culture medium was next added to each sample, after which cells were incubated for 4 h before imaging.

3 × 10^4^ MCF-10CA1h cells were allowed to attach to 8-well chambered cover-glass overnight, prior to transfection (Zhan et al., 2020). Cells were at ∼30% confluency at the time of transfection. Transient transfection of the cells with 80 ng each of pStargazin-GFP-LOVpep, p2XPDZ-mCherry-LARG (DH), or pLifeact-7-iRFP670 was performed using Lipofectamine 3000 following manufacturer’s instructions (Wagner and Glotzer, 2016; Padilla-Rodriguez et al., 2018). After 4 h, transfection medium was aspirated and cells were cultured in 500 µL fresh growth medium for 48 h before imaging.

### Optogenetic recruitment

All optogenetic experiments were done in the absence of any chemoattractant. Photoactivation was done with argon laser (488 nm excitation). All recruitable effectors, tagged with mCherry, RFP, or tdTomato were visualized with solid-state laser (561 nm excitation and 579–632 nm emission). miRFP703 or iRFP670 was excited with 633 nm diode laser and emission was collected at 659–709 nm. DIC was acquired using the T-PMT associated with red channel. 40X/1.30 Plan-Neofluar oil objective, along with digital zoom, was used. For imaging mammalian cells, both microscopes contained a temperature-controlled chamber held at 37°C and 5% CO_2_. Zeiss 800 or 780 was operated by ZEN Blue or Black software, respectively (Kuhn et al., 2021; Banerjee et al., 2022; Banerjee et al., 2023; Pal et al., 2023).

For photoactivation of differentiated HL-60 cells, we discovered that their pre-treatment with heat-killed *Klebsiella aerogenes* greatly improved efficiency of cryptochrome system. Briefly, 10^7^ differentiated neutrophils, grown on 10 cm culture dish, were incubated with 13 μg/mL heat-killed bacteria for 7 h. Next, cells were allowed to adhere to chambered coverglass, coated with fibronectin at a density of 35–40 μg/cm^2^, for 40 min. Dead bacteria and unattached neutrophils were rinsed off from fibronectin-coated surface before imaging. We performed global recruitment experiments on Zeiss LSM780 microscope. The 488 nm excitation laser was turned on after imaging for at least 5 min. Image acquisition and subsequent photoactivation were carried out once every 7 s. Laser intensity during image capture was maintained at a low level (laser power of 1.7%–2% or 0.14–0.17 W/cm^2^ at the objective) and exposure time was set at ∼2 s. This ensured RasGRP4 recruitment over the cell boundary without causing light damage. All local recruitment experiments were done using the Zeiss LSM800 microscope. A small region of interest was drawn near the cell (shown as dashed white boxes in the images), which was illuminated with the 488 nm laser (power of 5%–7% or 0.6–0.8 W/cm^2^ at the objective) in multiple iteration. All imaging was completed within 5 h (Pal et al., 2023).

Before imaging RAW 264.7 cells, culture medium was removed and 450 µL pre-warmed HBSS buffer was added to cells. For MCF-10CA1h cell imaging, culture medium was aspirated and replaced with 500 µL pre-warmed imaging medium. Image acquisition and photoactivation were carried out once every 7 s or 8 s for macrophage or MCF-10CA1h cells, respectively. Other parameters for recruitment studies were similar to HL-60 cells.

Vegetative and developed *Dictyostelium* cells were allowed to adhere on chambered coverglass for 30 min before imaging. For recruitment, 488 nm laser was switched on after imaging for at least 5–10 min. Photoactivation during single plane imaging was carried out once every 5–15 s since half-life of iLID-SspB was ∼30 s. Since *Dictyostelium* are light sensitive, very low laser intensity (0.017–0.030 W/cm^2^ or 0.06–0.08 W/cm^2^ at the objective on the Zeiss LSM780 or LSM800, respectively) was used to stably recruit SspB-RacGEF over the cell boundary without any light damage.

### Cell sorting

HL-60 cells, 5 days after infection, were harvested, washed twice and resuspended in sorting buffer at a density of 15 × 10^6^ cells/mL. Cells were declumped by passing suspension through 40 μm cell strainer once and collected in 5 mL round-bottom tubes. Wild type HL-60 cells (2 × 10^5^ cells resuspended in 300 µL sorting buffer) was used as unstained control. Sorting was done at the Ross Flow Cytometry Core and Centre for Cell Dynamics, JHU using 100 μm microfluidic sorting chip. 561 nm excitation laser was used to sort and collect RFP expressing cells, whereas 633 nm excitation was used to sort miRFP703 expressors. The detector has 735LP and 780/60BP. High expressors (top 1%–10%) were taken in 0.5–5 mL collection medium, spun down, and medium was discarded. Sorted cells were resuspended in fresh collection medium and grown to confluency.

### SDS-PAGE and western blotting

For more details, see Supplementary Materials and Methods.

### Data analysis

**FIGURE 2:**
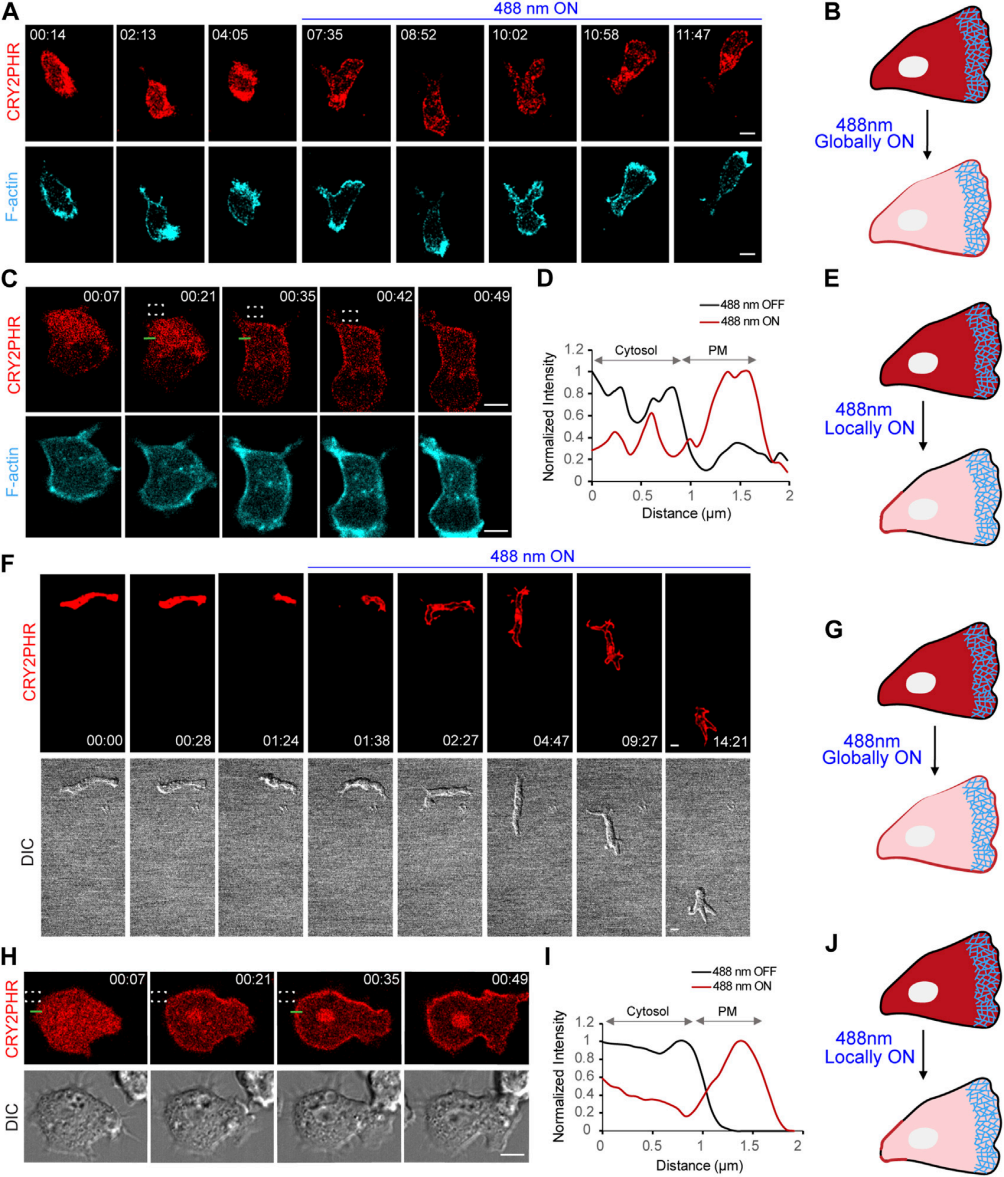
Establishment of cryptochrome system in neutrophils and *Dictyostelium*. **(A)** Time-lapse confocal images of differentiated HL-60 neutrophil expressing CRY2PHR-mCherry (red; upper panel) and LifeAct-miRFP703 (cyan; lower panel), before or after 488 nm laser was turned on globally. Time in min:sec format. Scale bars represent 5 μm. **(B)** Cartoon showing global recruitment of CRY2PHR-mCherry from cytosol to plasma membrane of neutrophils after turning on 488 nm laser globally. **(C)** Time-lapse images of differentiated HL-60 neutrophil expressing CRY2PHR-mCherry (red; upper panel) and LifeAct-miRFP703 (cyan; lower panel). CRY2PHR was recruited exclusively to the back of the neutrophil by applying 488 nm light near it, as denoted by dashed white box. Time in min:sec format. Scale bars represent 5 μm. **(D)** A linescan across the cytosol-membrane of the cell in (C; shown with green line) denoting increased CRY2PHR intensity on the membrane after laser was switched on near the region. **(E)** Cartoon showing local recruitment of CRY2PHR-mCherry from cytosol to the back membrane of neutrophils after turning on 488 nm laser locally. **(F)** Time-lapse confocal images of developed *Dictyostelium* expressing CRY2PHR-mCherry (red; upper panel) before or after 488 nm laser was switched on globally. Cell morphology and motility were visualized in DIC channel (lower panel). Time in min:sec format. Scale bars represent 5 μm. **(G)** Cartoon showing global recruitment of CRY2PHR-mCherry from cytosol to plasma membrane of *Dictyostelium* after turning on 488 nm laser globally. **(H)** Time-lapse images of developed *Dictyostelium* expressing CRY2PHR-mCherry (red; upper panel). CRY2PHR was recruited exclusively to the back of the cell by applying 488 nm light near it, as denoted by dashed white box. Cell morphology and protrusive activity were visualized in DIC channel (lower panel). Time in min:sec format. Scale bars represent 5 μm. **(I)** A linescan across the cytosol-membrane of the cell in (H; shown with green line) denoting increased CRY2PHR intensity on the membrane after laser was switched on near the region. **(J)** Cartoon showing local recruitment of CRY2PHR-mCherry from cytosol to the back membrane of *Dictyostelium* after turning on 488 nm laser locally.

**FIGURE 3:**
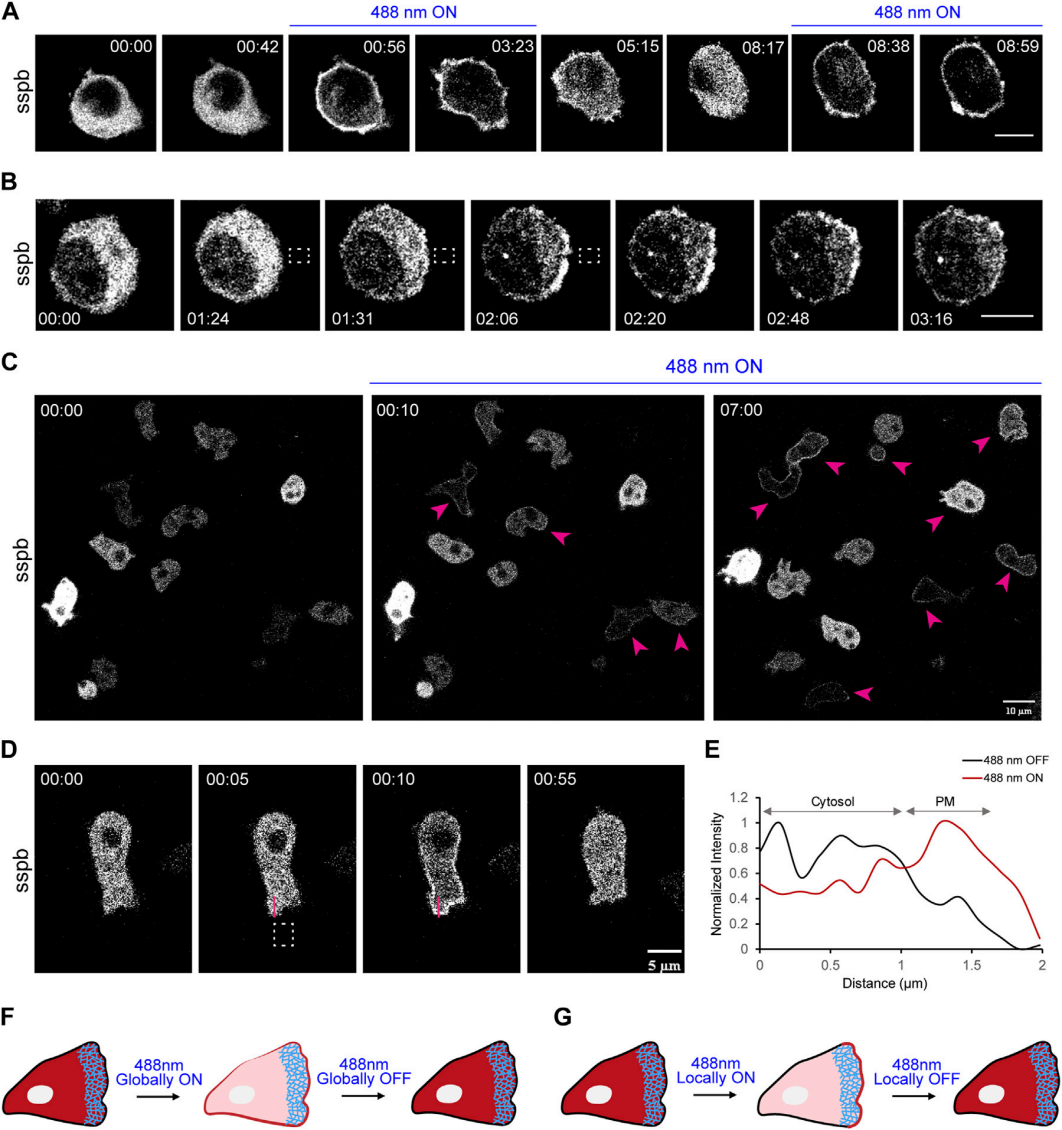
Establishment of LOV domain-based iLID system in macrophages and *Dictyostelium*. **(A)** Time-lapse confocal images of RAW 264.7 macrophage expressing tgRFPt-SSPB R73Q (or sspb) before or after 488 nm laser was turned on globally. Time in min:sec format. Scale bars represent 5 μm. **(B)** Time-lapse images of RAW 264.7 macrophage expressing tgRFPt-SSPB R73Q (or sspb) which was recruited exclusively to one side of the cell by applying 488 nm light near it, as denoted by dashed white box. Both **(A, B)** highlight the fast reversibility of this system. Time in min:sec format. Scale bars represent 5 μm. **(C)** Time-lapse confocal images of a field of vegetative *Dictyostelium* expressing tgRFPt-SSPB R73Q (or sspb) before or after 488 nm laser was switched on globally. Pink arrows denote successful recruitment in cells. Time in min:sec format. Scale bars represent 5 μm. **(D)** Time-lapse images of vegetative *Dictyostelium* expressing tgRFPt-SSPB R73Q (or sspb) which was recruited exclusively to one side of the cell by applying 488 nm light near it, as denoted by dashed white box. Time in min:sec format. Scale bars represent 5 μm. **(E)** A linescan across the cytosol-membrane of the cell in **(D)** denoting increased sspb intensity on the membrane after laser was switched on near the region. **(F)** Schematic representation of experimental data shown in **(A)**. **(G)** Schematic representation of experimental data shown in **(B, D)**. Both **(F, G)** highlight fast reversibility of iLID system with both global or local recruitment experiments.

For Figure 1, Figure 2D, Figure 2I, Figure 3E, Figure 5D, and Supplementary Figure S6B, linescans were created on Fiji/ImageJ 1.52i software (https://imagej.nih.gov/ij/) (Schneider et al., 2012). On the red channel, a “straight line” segment (12-pixel width) was drawn across the cell using the ‘line tool’ option. Next, we obtained the average intensity value along that line using the “Plot Profile” option. Values were normalized and graphed in Microsoft Excel.

For Supplementary Figure S3C, turn-off rate (membrane-cytosol cycling) for the mutants was calculated by counting the number of frames from the last flash of the laser till the signal in the red channel was no longer present on the cell boundary. The frame with the last flash of the 488 nm laser was considered as “0 s” and frame rate was 7 s. For Supplementary Figure S3E, red signal intensity in the cytosol and nucleus was measured immediately before or after turning on the laser. This allowed us to calculate percentage decrease in signal intensity in the cytosol/nucleus with photoactivation. We used these values to obtain the percentage of total recruitable protein translocated to membrane for each cell. GraphPad Prism 8 was used to prepare box-and-whisker plots.

For Supplementary Figure S4B, acquired images from different experimental conditions were adjusted to the same contrast by ImageJ software. Cells were outlined by “polygon tool” in ImageJ and the mean intensity for each was recorded. GraphPad Prism 8 (https://www.graphpad.com/) was next used to prepare the scatter plot.

**FIGURE 4:**
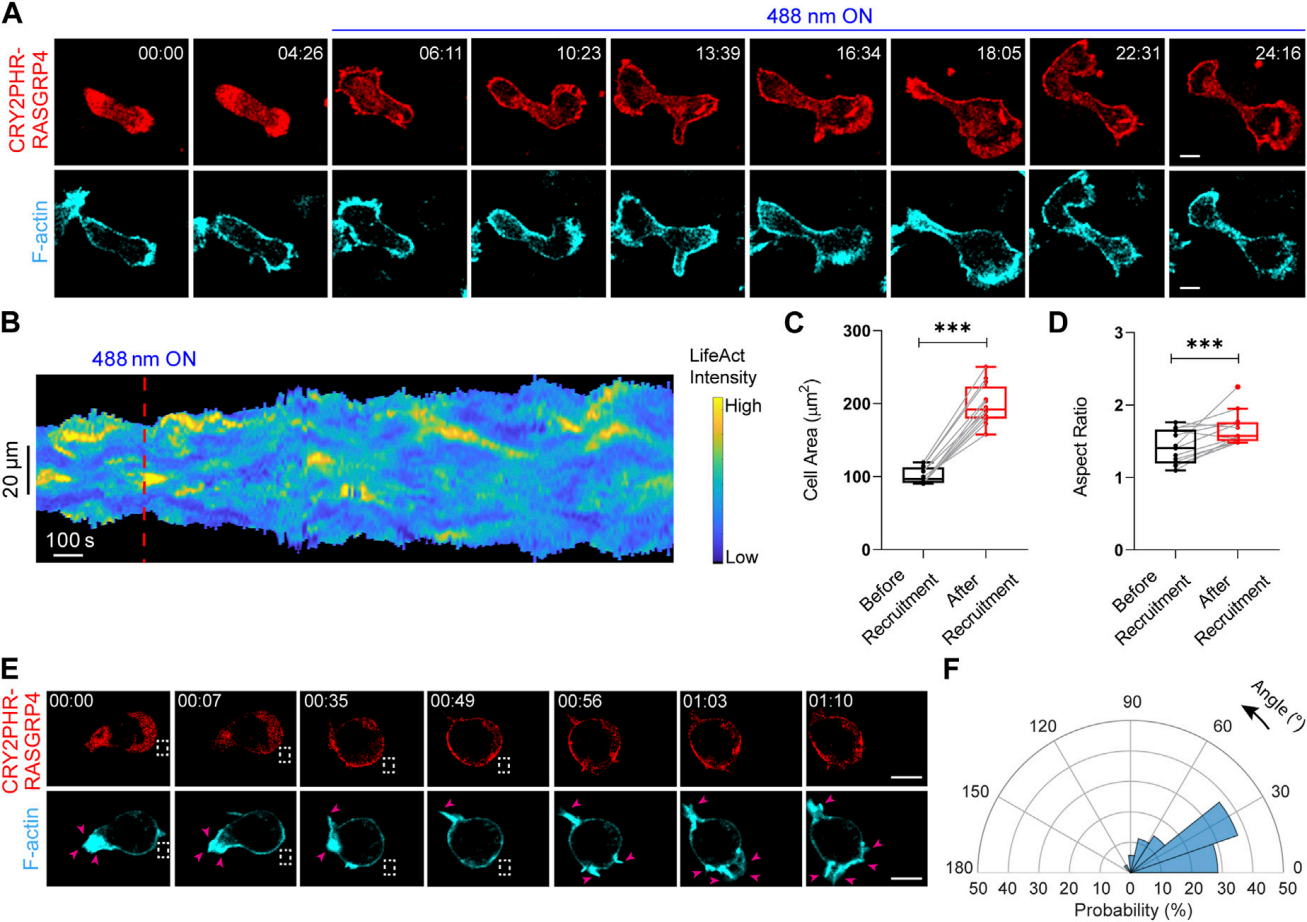
Establishment of an opto-RasGEF system in neutrophils. **(A)** Time-lapse confocal images of differentiated HL-60 neutrophil expressing CRY2PHR-mCherry-RasGRP4 (red; upper panel) and LifeAct-miRFP703 (cyan; lower panel), before or after 488 nm laser was turned on globally. Time in min:sec format. Scale bars represent 5 μm. **(B)** Representative membrane kymograph of cortical LifeAct intensity in opto-RasGEF expressing neutrophil before or after 488 nm laser was turned on. The linear color map denotes blue is the lowest LifeAct intensity and yellow is the highest. Duration of the kymograph is 29 min. Box-and-whisker plots of **(C)** cell area and **(D)** aspect ratio, before or after RasGEF recruitment. n_c_ = 12 from atleast three independent experiments. Asterisks denote significant difference, ***p ≤ 0.001 (Wilcoxon-Mann-Whitney rank-sum test) **(E)** Time-lapse confocal images of differentiated HL-60 neutrophil expressing CRY2PHR-mCherry-RasGRP4 (red; upper panel) and LifeAct-miRFP703 (cyan; lower panel). Opto-RasGEF was recruited precisely to the back of the cell as shown with the dashed white box, resulting in new F-actin protrusions (shown by pink arrows in LifeAct panel) at the recruitment site. Time in min:sec format. Scale bars represent 5 μm. **(F)** Polar histogram of opto-RasGEF (n_c_ = 14 and n_p_ = 35) demonstrate greater probability of new protrusion generation near the recruitment region.

For Figure 4B, cell was first segmented against the background with help of a custom code written in MATLAB 2019b (https://www.mathworks.com/). Next, membrane kymograph was created from the segmented cell as described previously (Banerjee et al., 2022; Banerjee et al., 2023; Pal et al., 2023). A linear color map for normalized intensities was used; blue denoted the lowest intensity whereas yellow denoted the highest.

For Figures 4C,D, and Figures 6B–D, cells were segmented in Fiji/ImageJ 1.52i software (Pal et al., 2023). This was done in a stepwise manner. First, using the “Threshold” option, the image stack was thresholded. We made sure that the “Calculate threshold for each image” box was kept unchecked, and the range was not reset. Second, using the “Analyzed Particles” option, we created cell masks by size-based thresholding. Third, these binary masks were optimized by performing “Fill holes”, “Dilate”, and “Erode” multiple times. Fourth, “Area”, “Shape descriptors”, “Centroid”, “Min and max gray value” and “Mean gray value” boxes in the “Set Measurements” tab under “Analyze” were checked. This allowed us to obtain values for aspect ratio and centroid coordinates. Using GraphPad Prism 8, mean and SEM obtained from aspect ratio value replicates were plotted. We calculated velocity by quantifying displacement between two consecutive frames. Subsequently, cell speed was acquired by dividing displacement with time interval. Using GraphPad Prism 8, average cell speed values, obtained from time-averaging cell speeds over all the frames, were plotted as box-and-whisker plots.

For Figure 4F and Figure 6, local protrusion formation was analyzed in a stepwise manner (Banerjee et al., 2022; Pal et al., 2023). First, using the “segmented line” tool in Fiji/ImageJ software, GEF recruitment region in the red channel was marked. Second, the midpoint of the recruited region was determined with the sequential use of customized macros, “Fit Spline” and “Straighten”. Third, after having determined the centroid with assistance from another Fiji/ImageJ macro, the angle between the nascent protrusion and recruitment region midpoint was calculated using the “angle” tool, holding the centroid as vertex. Using “polarhistogram” in MATLAB, these values were plotted. Atleast 35 fresh protrusions were considered for each histogram, and the minimum number of bins for each plot was calculated by Sturges’ formula.

**FIGURE 5:**
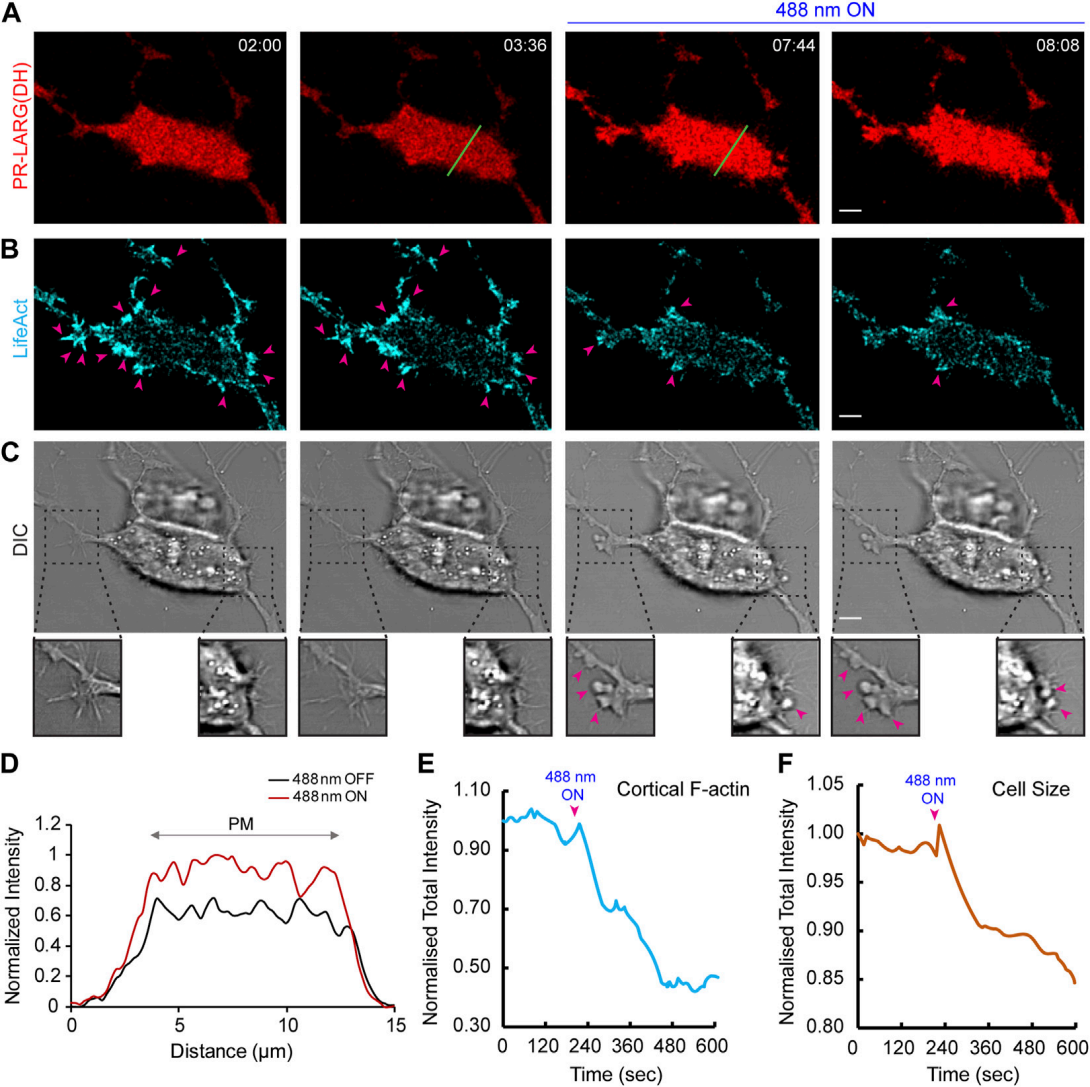
Establishment of an opto-RhoGEF system in epithelial cells. Time-lapse confocal images of MCF-10CA1h cell expressing 2XPDZ-mCherry-LARG (DH) (red; **(A)** and Lifeact-7-iRFP670 (cyan); **(B)**, before or after 488 nm laser was turned on globally. Confocal slices are focused at the substrate-attached basal surface of the cell. 488 nm light was turned on at 03:44 (min:sec), and images were acquired every 8 s. Pink arrows denote F-actin rich protrusions in the cell, and its subsequent decrease after laser was switched on. Cell morphology and protrusions were visualized in the DIC channel **(C)**. Disappearance of protrusions and appearance of blebbing after recruitment can be visualized in magnified view with pink arrows. Time in min:sec format. Scale bars represent 5 μm. **(D)** A linescan across the bottom surface of the cell in (A; shown with green line) denoting uniform increase in LARG (DH) intensity on the cell membrane after laser was switched on globally. Quantifications display reduction in cortical F-actin **(E)** and decrease in cell size **(F)** upon opto-RhoGEF recruitment to the cell membrane.

In Figures 5E,F, cell was binarized using ImageJ based on the LifeAct channel. One typical Life-act labelled actin patch, as indicated by pink arrows in Figure 5B, was quantified throughout the video to show the change of newly formed cortical F-actin. Graphs were plotted using Microsoft Excel.

### Statistical analysis

**FIGURE 6:**
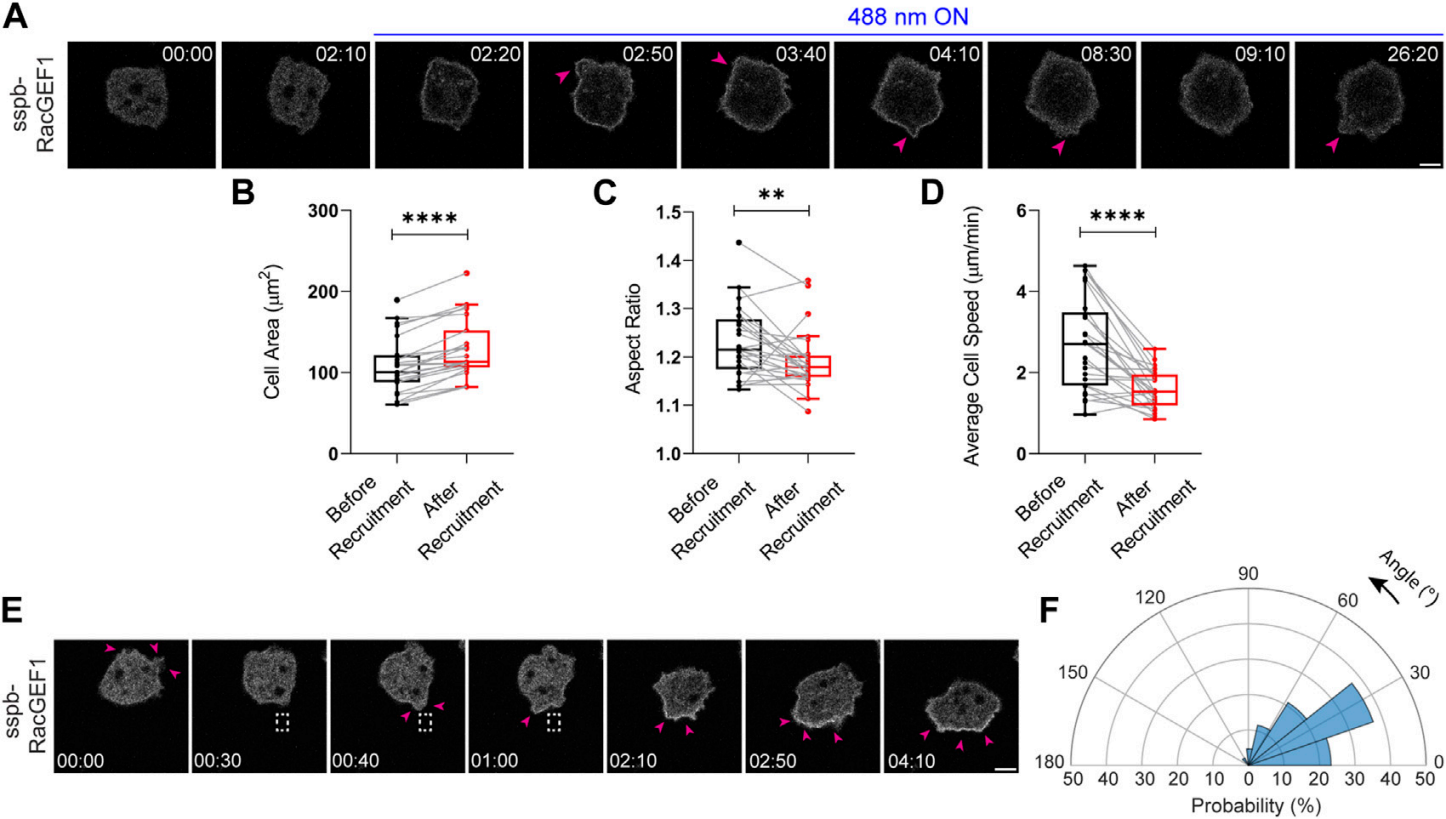
Establishment of an opto-RacGEF system in *Dictyostelium*. **(A)** Time-lapse confocal images of vegetative *Dictyostelium* cell expressing tgRFPt-SspB R73Q-RacGEF1 (catalytic domain) before or after 488 nm laser was turned on globally. Pink arrows denote appearance of protrusions on the cell periphery after opto-RacGEF recruitment. Time in min:sec format. Scale bars represent 5 μm. Box-and-whisker plots of **(B)** cell area, **(C)** aspect ratio and **(D)** average speed, before or after RacGEF recruitment. n_c_ = 25 from atleast three independent experiments. Asterisks denote significant difference, **p ≤ 0.01 and ****p ≤ 0.0001 (Wilcoxon-Mann-Whitney rank-sum test) **(E)** Time-lapse confocal images of vegetative *Dictyostelium* expressing opto-RacGEF which was recruited precisely to the back of the cell as shown with the dashed white box. This resulted in new protrusions (shown by pink arrows) at the recruitment site causing the cell to move towards the direction of the light. Time in min:sec format. Scale bars represent 5 μm. **(F)** Polar histogram of opto-RacGEF (n_c_ = 35 and n_p_ = 52) demonstrate greater probability of new protrusion generation near the recruitment region.

All statistical analyses were done on GraphPad Prism 8 software. For Supplementary Figures S3D-E, unpaired 2-tailed non-parametric test (Mann-Whitney test) was done. For Figures 4C,D, Figures 6B–D, and Supplementary Figure S4B, paired 2-tailed non-parametric test (Wilcoxon matched-pairs signed rank test) was used. Results are expressed as mean ± SD from at least 3 independent experiments. ns denotes *p* > 0.05, ** denotes *p* ≤ 0.01, *** denotes *p* ≤ 0.001, **** denotes *p* ≤ 0.0001.

## Results

### Establishment of different cryptochrome and iLID optogenetic systems in multiple cell lines

In this study, we developed and utilized blue light-inducible dimerization systems to acutely perturb Ras superfamily GEFs in migrating cells. As discussed in the methods section, we generated stable cell lines, co-expressing cryptochrome optogenetic system with F-actin biosensor, LifeAct-miRFP703, in differentiated HL-60 neutrophils. Interestingly, western blot and confocal imaging results showed that treating these triple expressors with heat-killed bacteria, before experimentation, improved expression levels of CRY2PHR recruitable component as well as LifeAct, in a time-dependent manner (Supplementary Figures S1A,B). We presume that this pre-treatment would have a similar effect on expression of the membrane anchor, CIBN-CAAX, but we could not test that in our study. When we turned on the 488 nm laser on the entire cell, it caused cytosolic CRY2PHR to recruit globally on the plasma membrane within seconds. LifeAct expression strongly defined the front of these migrating cells (Figures 2A,B).

Could we spatially confine cryptochrome recruitment to a certain region on the neutrophil membrane? To answer this, we selectively turned on the blue light at the back of the cell, as denoted by the dashed white box. Time lapse imaging and linescan analysis clearly showed that nearly half of the total cytosolic CRY2PHR protein translocated to the illuminated region of the membrane within 20 s (Figures 2C–E). This result led us to ask whether the distance of the illuminated region (dashed white box) from the cell boundary would have any effect on localized recruitment. When the laser was directed far from the cell boundary, we did not observe any appreciable recruitment (Supplementary Figure S2A). Once the blue light was applied near the boundary, CRY2PHR recruitment was strongly localized near the illumination region (Figure 2C; Supplementary Figure S2B). However, when we shined the laser within the cell boundary, it led to global recruitment of the CRY2PHR protein (Supplementary Figure S2C). These data suggest that blue light is travelling past the edges of the region of illumination, upto a certain distance, leading to unwanted recruitment. Hence, we performed all localized illumination experiments by applying blue light just outside the cell boundary.

With our success in neutrophils, we attempted to establish the CRY2-CIBN system in *Dictyostelium* amoeba. We were successfully able to express CRY2PHR (red) and CIBN-CAAX (untagged) in developed and vegetative *Dictyostelium* as demonstrated by our global and local recruitment experiments, respectively (Figures 2F,H). CRY2PHR recruitment was fairly uniform upon global illumination with blue light, whereas it could be spatially constricted to a specific part of the cell membrane by localized irradiation (Figures 2F–J). Moreover, light-sensitive *Dictyostelium* seemed to be unaffected with the blue laser as suggested by their polarized morphology and motility (Figures 2F,H).

To aid our investigation, we characterized several fast-cycling cryptochrome mutants. In RAW 264.7 macrophages, both CRY2PHR (W349R)-mCherry and CRY2PHR (R489E, A491D)-tdTomato mutants translocated back to the cytosol within 3 min and 4.5 min of switching off the blue laser, respectively. This is a significant improvement from the turn-off rate of ∼9.5 min for CRY2PHR-mCherry (Supplementary Figures S3A–D). Moreover, 40% of total CRY2PHR (R489E, A491D)-tdTomato mutant translocated to the membrane after irradiation, compared to 21% or 23% for CRY2PHR-mCherry or CRY2PHR (W349R)-mCherry, respectively (Supplementary Figure S3E). The primary reason for this is that a major portion of total CRY2PHR-mCherry or CRY2PHR (W349R)-mCherry localized within the nucleus upon expression, whereas CRY2PHR (R489E, A491D)-tdTomato expressed entirely in the cytosol and was excluded from the nucleus (Supplementary Figures S3A–C). Thus, we identified CRY2PHR (R489E, A491D)-tdTomato mutant to be the most recruitable cryptochrome with a relatively fast membrane-cytosol turnover, making it ideal for studying time-critical biological reactions.

In addition to the CRY2-CIBN system, we established an improved Light Induced Dimerization (iLID) optogenetic system in mammalian and amoeba cells to examine GEF function. As shown in Figures 3A,F, SspB component was optically recruited uniformly on the macrophage cell membrane, and soon after switching off the light source, translocated back to the cytosol. SspB could be subsequently recruited after switching on the laser again. With localized illumination, as shown with the dashed white box, we noticed a distinct crescent of recruited SspB on the membrane, which gradually diminished within a minute of taking away the light (Figures 3B,G). In *Dictyostelium,* we integrated SspB with a doxycycline-inducible expression system under the inducible promoter, TRE-P_min_, which consists of seven repeats of the TetO operator fused to a small fragment of the act15 promoter (Veltman et al., 2009). This allowed us to tightly control expression of recruitable SspB in a time-dependent manner upto 14 h, making this a useful tool for preventing build-up of potentially toxic effector proteins for optical studies (Supplementary Figures S4A,B). Irradiating 488 nm laser on these doxycycline-treated cells elicited uniform membrane recruitment within 10 s (middle panel; Figure 3C). With prolonged exposure to light, we observed recruitment in more cells in the population (right panel; Figure 3C). When we restricted the laser at a certain location, it induced a gradient of recruited SspB near the illuminated membrane. Once this was turned off, SspB again translocated back to the cytosol (Figures 3D,G). Linescan analysis clearly underlines SspB recruitment and shows that ∼50% of total cytosolic SspB was recruited to the membrane (Figure 3E). Moreover, upon testing SspB recruitability with varying blue laser strengths (0.2%–5% of total laser intensity or 0.024–0.639 W/cm^2^ laser power density), we identified a laser power density of ∼0.061 W/cm^2^ (0.5%) to be ideal (Supplementary Figure S5C); a weaker laser strength was unable to efficiently recruit SspB (Supplementary Figures S5A, B) whereas higher intensities compelled *Dictyostelium* to move away from the light source (Supplementary Figures S5D,E). These results suggested easy recruitability and high membrane-cytosol turnover of the iLID system. Overall, both CRY2-CIBN and iLID-SspB systems provided us with tightly-regulated, spatiotemporal control necessary to investigate GEF function in different cell types.

### Optical recruitment of different GEF proteins has strong effect on cytoskeletal dynamics, cell shape and motility

We first examined the effects of an opto-RasGEF in neutrophil morphology and migration (Pal et al., 2023). Blue light-driven global recruitment of full-length RasGRP4 resulted in increased spreading and F-actin patches in these cells (Figure 4A). A representative membrane kymograph showed increase in cell area with a concomitant increase in LifeAct patches after RasGRP4 recruitment (Figure 4B). Across the population, RasGRP4 recruitment induced a ∼90% or ∼20% increase in neutrophil cell area or aspect ratio (which serves as a proxy for cell polarity), respectively (Figures 4C,D). Next, selectively recruiting RasGRP4 to the back of the cell, as shown by dashed white box, caused new protrusions to arise locally with a concomitant disappearance of mature protrusions from the other end (Figure 4E). Angular histogram analysis showed that probability of fresh protrusion formation is highest at or near the RasGRP4 recruitment site (Figure 4F). Thus, opto-RasGRP4 increased cell area, F-actin polymerization, and reversed pre-exiting polarity. Since RasGEFs activate small Ras GTPase by exchanging GDP to GTP, we hypothesized that cytoskeletal effects induced by opto-RasGRP4 should be similar to an optically-recruited constitutively active Ras, KRas4B G12V. Upon locally recruiting CAAX-deleted KRas4B G12V to the *Dictyostelium* membrane*,* we noticed new protrusions appearing at the recruitment site (denoted by pink arrow in Supplementary Figure S6).

We next investigated how optically recruiting the catalytic Dbl homology (DH) domain of LARG would affect cell shape or size (O'Neill et al., 2018; Wagner and Glotzer, 2016; Rich et al., 2020; Inaba et al., 2021; Valon et al., 2017; Ridley, 2015). During imaging, we focused near the substrate-attached bottom surface of the epithelial cell as opposed to a middle section of the cell, as shown previously. Time-lapse imaging and linescan analysis demonstrated that the red fluorescence intensity of the LARG DH domain increased by ∼30% on the bottom surface of the MCF-10CA1h cell, after blue light was switched on, suggesting its recruitment from cytosol to the membrane (Figures 5A,D). This reduced the number of distinct F-actin patches at the cell cortex as denoted by pink arrows in Figure 5B. Overall, there was a >50% reduction in cortical F-actin intensity upon LARG (DH) recruitment (Figure 5E). This caused increased blebbing (shown with pink arrows in Figure 5C) and cell shrinkage (Figure 5F).

We next focused on opto-RacGEF mediated Rac GTPase activation and its role in regulating actin cytoskeleton and cell movement (Park et al., 2004; Pankov et al., 2005; Wu et al., 2009; Wang et al., 2010; Yoo et al., 2010; Kato et al., 2014). Upon global recruitment of the GEF domain of RacGEF1, the *Dictyostelium* cell triggered more protrusions around its periphery, as shown by pink arrows, and demonstrated appreciable spreading (Figure 6A). Across the population, RacGEF1 recruitment resulted in ∼20% increase in cell area (Figure 6B). There was a concomitant ∼10% or ∼40% reduction in cell polarity or average speed, respectively (Figures 6C,D). Next, we looked at the local effects of RacGEF1 recruitment on cell migration. Since RacGEFs trigger actin polymerization usually at the front of migrating cells, we anticipated that locally recruiting RacGEF1 catalytic domain would either enforce or oppose the pre-existing direction of migration. Indeed, when we locally recruited RacGEF1 to the back of the migrating cell, it first induced a protrusion at the recruitment site (denoted by pink arrows near the dashed white box), then changed the direction of migration by generating bigger membrane ruffles, and finally made the cell move towards the light source (Figure 6E). Angular histogram analysis confirmed that the probability of new protrusion generation was greatest at or near the site of GEF recruitment (Figure 6F).

## Discussion

In the last decade, we have witnessed a meteoric increase in size of the optogenetic toolkit for inducing specific and acute perturbations in signal transduction and cytoskeletal networks. This technology has enabled scientists to inactivate or activate particular biochemical activities and interactions with increasingly superior spatiotemporal resolution in live-cell experiments. Wider applications of these tools in complex tissue and animal systems will require technological improvements, specifically towards fine tuning key parameters of binding affinity and kinetics, and developing new proteins with red-shifted excitation spectra which offer compatibility with two-photon imaging. Moreover, current optical systems depend on overexpression of individual proteins for their functioning, which makes it hard to control signaling activity at endogenous expression levels. Despite these shortcomings, optogenetics has become the most useful tool in basic and biomedical research, as highlighted by Nature Methods as ‘Method of the Year’ in 2010 (Ross et al., 2016; Repina et al., 2017; Kuhn et al., 2021).

Here, we have successfully utilized these optical tools and demonstrated that multiple light-induced dimerization systems can be transferred to various cell types, ranging from immune and epithelial cells to soil amoeba. We have specifically validated two blue light-triggered optogenetic systems, CRY2-CIBN and iLID, in these cells. Of these two, the cryptochrome-based system was compatible in all cell lines we tested; however, we were unable to establish a functional iLID system in developed *Dictyostelium* and HL-60 neutrophil-like cells. This was primarily due to poor expression and mislocalization of iLID protein components in these cells. However, instantaneous uncoupling of iLID dimers upon light removal allowed a finer temporal control than cryptochrome. Coupled with a tendency for low cytosolic clustering in the light state, iLID was superior to the cryptochrome system which displayed high light-dependent CRY2–CRY2 homo-oligomerization. To improve upon these shortcomings, we tested various available CRY2 mutants of which CRY2(W349R) fused to mCherry and CRY2 (R489E, A491D) fused to tandem dimeric Tomato (tdTomato) were the most promising (Taslimi et al., 2016; Duan et al., 2017). Both mutants showed significantly faster dark-state membrane-cytosol cycling rates, comparable to the iLID system. On account of low light-dependent cytosolic CRY2 clustering, the CRY2 (R489E, A491D)-tdTomato mutant was more recruitable on the plasma membrane than all other CRY2 fusion proteins. It would be interesting to check if a combination of these two mutants, i.e., a CRY2(W349R, R489E, A491D)-tdTomato, would display a faster dark-state cycling rate with a higher membrane recruitability.

We tested the physiological potential of our optogenetic systems by acutely perturbing membrane activity of GEFs specific for Ras, Rho, and Rac GTPases, which are important regulatory components of growth, metabolic, and migration signaling (Pylayeva-Gupta et al., 2011; Goicoechea et al., 2014; Devreotes et al., 2017; Lawson and Ridley, 2018; Pal et al., 2019; Li et al., 2020). We observed that opto-GEFs could globally or locally stimulate GTPase signaling, resulting in cytoskeletal reorganization and profound changes in cell shape and size. Global recruitment of opto-RasGEF and -RacGEF resulted in cell spreading whereas their local recruitment to the cell back caused neutrophil and *Dictyostelium* cells to form new protrusions at the recruitment site. With opto-RacGEF, we were even able to induce directional migration in the amoeba. On the other hand, opto-RhoGEF caused epithelial cells to shrink in size and form blebs around the periphery (Yoo et al., 2010; O'Neill et al., 2018; Wu et al., 2009; Wu et al., 2011; Pal et al., 2023; Kato et al., 2014; Wang et al., 2010; Ridley, 2015; Pankov et al., 2005; Coleman and Olson, 2002). All of these alterations could be directly induced, by recruiting either full length GEF proteins or only their GEF domains, within a matter of minutes without allowing signaling or cytoskeletal networks to readjust. Thus, optical control of signaling is an effective approach for spatiotemporal control of cellular signaling (Schwechter et al., 2013; Toettcher et al., 2013; Guntas et al., 2015; Valon et al., 2017; Yang et al., 2018; Lamas et al., 2020; Inaba et al., 2021; De Belly et al., 2023). Opto-SOS, a recruitable RasGEF, induced ERK signaling in *Drosophila* and uncovered the role of this pathway in promoting endodermal differentiation (Toettcher et al., 2013; Krueger et al., 2019; McFann et al., 2021). An opto-Rho1 (DH domain of LARG) induced ectopic deformations in the ventral and dorsal epithelia of *Drosophila* embryos (Rich et al., 2020). In light of these elegant studies, our opto-GEFs will be a beneficial addition to the optogenetic toolbox to probe mechanisms determining cell fate during embryogenesis.

## Data Availability

The original contributions presented in the study are included in the article/Supplementary Material, further inquiries can be directed to the corresponding authors.

## References

[B1] Artemenko Y. Lampert T. J. Devreotes P. N. (2014). Moving towards a paradigm: Common mechanisms of chemotactic signaling in Dictyostelium and mammalian leukocytes. Cell. Mol. Life Sci. 71, 3711–3747. 10.1007/s00018-014-1638-8 24846395 PMC4162842

[B2] Banerjee T. Biswas D. Pal D. S. Miao Y. Iglesias P. A. Devreotes P. N. (2022). Spatiotemporal dynamics of membrane surface charge regulates cell polarity and migration. Nat. Cell. Biol. 24, 1499–1515. 10.1038/s41556-022-00997-7 36202973 PMC10029748

[B3] Banerjee T. Matsuoka S. Biswas D. Miao Y. Pal D. S. Kamimura Y. (2023). A dynamic partitioning mechanism polarizes membrane protein distribution. bioRxiv. 10.1101/2023.01.03.522496 PMC10689845 38036511

[B4] Bell G. R. R. Rincon E. Akdogan E. Collins S. R. (2021). Optogenetic control of receptors reveals distinct roles for actin- and Cdc42-dependent negative signals in chemotactic signal processing. Nat. Commun. 12, 6148. 10.1038/s41467-021-26371-z 34785668 PMC8595684

[B5] Bos J. L. Rehmann H. Wittinghofer A. (2007). GEFs and GAPs: Critical elements in the control of small G proteins. Cell. 129, 865–877. 10.1016/j.cell.2007.05.018 17540168

[B6] Cherfils J. Zeghouf M. (2013). Regulation of small GTPases by GEFs, GAPs, and GDIs. Physiol. Rev. 93, 269–309. 10.1152/physrev.00003.2012 23303910

[B7] Coleman M. L. Olson M. F. (2002). Rho GTPase signalling pathways in the morphological changes associated with apoptosis. Cell. Death Differ. 9, 493–504. 10.1038/sj.cdd.4400987 11973608

[B8] de Beco S. Vaidziulyte K. Manzi J. Dalier F. di Federico F. Cornilleau G. (2018). Optogenetic dissection of Rac1 and Cdc42 gradient shaping. Nat. Commun. 9, 4816. 10.1038/s41467-018-07286-8 30446664 PMC6240110

[B9] De Belly H. Yan S. Borja da Rocha H. Ichbiah S. Town J. P. Zager P. J. (2023). Cell protrusions and contractions generate long-range membrane tension propagation. Cell. (bioRxiv preprint). 10.1016/j.cell.2023.05.014 PMC10330871 37311454

[B10] Devreotes P. N. Bhattacharya S. Edwards M. Iglesias P. A. Lampert T. Miao Y. (2017). Excitable signal transduction networks in directed cell migration. Annu. Rev. Cell. Dev. Biol. 33, 103–125. 10.1146/annurev-cellbio-100616-060739 28793794 PMC5792054

[B11] Ding Z. Dhruv H. Kwiatkowska-Piwowarczyk A. Ruggieri R. Kloss J. Symons M. (2018). PDZ-RhoGEF is a signaling effector for TROY-induced glioblastoma cell invasion and survival. Neoplasia 20, 1045–1058. 10.1016/j.neo.2018.08.008 30219706 PMC6140379

[B12] Duan L. Hope J. Ong Q. Lou H. Y. Kim N. McCarthy C. (2017). Understanding CRY2 interactions for optical control of intracellular signaling. Nat. Commun. 8, 547. 10.1038/s41467-017-00648-8 28916751 PMC5601944

[B13] Dull T. Zufferey R. Kelly M. Mandel R. J. Nguyen M. Trono D. (1998). A third-generation lentivirus vector with a conditional packaging system. J. Virol. 72, 8463–8471. 10.1128/JVI.72.11.8463-8471.1998 9765382 PMC110254

[B14] El-Brolosy M. A. Kontarakis Z. Rossi A. Kuenne C. Gunther S. Fukuda N. (2019). Genetic compensation triggered by mutant mRNA degradation. Nature 568, 193–197. 10.1038/s41586-019-1064-z 30944477 PMC6707827

[B15] El-Brolosy M. A. Stainier D. Y. R. (2017). Genetic compensation: A phenomenon in search of mechanisms. PLoS Genet. 13, 1006780. 10.1371/journal.pgen.1006780 PMC5509088 28704371

[B16] Francis S. A. Shen X. Young J. B. Kaul P. Lerner D. J. (2006). Rho GEF Lsc is required for normal polarization, migration, and adhesion of formyl-peptide-stimulated neutrophils. Blood 107, 1627–1635. 10.1182/blood-2005-03-1164 16263795 PMC1895409

[B17] Gaudet P. Fey P. Basu S. Bushmanova Y. A. Dodson R. Sheppard K. A. (2011). dictyBase update 2011: web 2.0 functionality and the initial steps towards a genome portal for the Amoebozoa. Nucleic Acids Res. 39, D620–D624. 10.1093/nar/gkq1103 21087999 PMC3013695

[B18] Goicoechea S. M. Awadia S. Garcia-Mata R. (2014). I'm coming to GEF you: Regulation of RhoGEFs during cell migration. Cell. Adh Migr. 8, 535–549. 10.4161/cam.28721 25482524 PMC4594598

[B19] Gray J. L. von Delft F. Brennan P. E. (2020). Targeting the small GTPase superfamily through their regulatory proteins. Angew. Chem. Int. Ed. Engl. 59, 6342–6366. 10.1002/anie.201900585 30869179 PMC7204875

[B20] Guntas G. Hallett R. A. Zimmerman S. P. Williams T. Yumerefendi H. Bear J. E. (2015). Engineering an improved light-induced dimer (iLID) for controlling the localization and activity of signaling proteins. Proc. Natl. Acad. Sci. U. S. A. 112, 112–117. 10.1073/pnas.1417910112 25535392 PMC4291625

[B21] Hadjitheodorou A. Bell G. R. R. Ellett F. Shastry S. Irimia D. Collins S. R. (2021). Directional reorientation of migrating neutrophils is limited by suppression of receptor input signaling at the cell rear through myosin II activity. Nat. Commun. 12, 6619. 10.1038/s41467-021-26622-z 34785640 PMC8595366

[B22] Housden B. E. Muhar M. Gemberling M. Gersbach C. A. Stainier D. Y. Seydoux G. (2017). Loss-of-function genetic tools for animal models: Cross-species and cross-platform differences. Nat. Rev. Genet. 18, 24–40. 10.1038/nrg.2016.118 27795562 PMC5206767

[B23] Idevall-Hagren O. Dickson E. J. Hille B. Toomre D. K. De Camilli P. (2012). Optogenetic control of phosphoinositide metabolism. Proc. Natl. Acad. Sci. U. S. A. 109, E2316–E2323. 10.1073/pnas.1211305109 22847441 PMC3435206

[B24] Inaba H. Miao Q. Nakata T. (2021). Optogenetic control of small GTPases reveals RhoA mediates intracellular calcium signaling. J. Biol. Chem. 296, 100290. 10.1016/j.jbc.2021.100290 33453281 PMC7949103

[B25] Insall R. H. Borleis J. Devreotes P. N. (1996). The aimless RasGEF is required for processing of chemotactic signals through G-protein-coupled receptors in Dictyostelium. Curr. Biol. 6, 719–729. 10.1016/s0960-9822(09)00453-9 8793298

[B26] Karunarathne W. K. Giri L. Patel A. K. Venkatesh K. V. Gautam N. (2013). Optical control demonstrates switch-like PIP3 dynamics underlying the initiation of immune cell migration. Proc. Natl. Acad. Sci. U. S. A. 110, E1575–E1583. 10.1073/pnas.1220755110 23569254 PMC3637758

[B27] Kato T. Kawai K. Egami Y. Kakehi Y. Araki N. (2014). Rac1-dependent lamellipodial motility in prostate cancer PC-3 cells revealed by optogenetic control of Rac1 activity. PLoS One 9, 97749. 10.1371/journal.pone.0097749 PMC4029798 24848679

[B28] Kennedy M. J. Hughes R. M. Peteya L. A. Schwartz J. W. Ehlers M. D. Tucker C. L. (2010). Rapid blue-light-mediated induction of protein interactions in living cells. Nat. Methods 7, 973–975. 10.1038/nmeth.1524 21037589 PMC3059133

[B29] Kok F. O. Shin M. Ni C. W. Gupta A. Grosse A. S. van Impel A. (2015). Reverse genetic screening reveals poor correlation between morpholino-induced and mutant phenotypes in zebrafish. Dev. Cell. 32, 97–108. 10.1016/j.devcel.2014.11.018 25533206 PMC4487878

[B30] Krueger D. Izquierdo E. Viswanathan R. Hartmann J. Pallares Cartes C. De Renzis S. (2019). Principles and applications of optogenetics in developmental biology. Development 146, 175067. 10.1242/dev.175067 PMC6914371 31641044

[B31] Kruger P. Saffarzadeh M. Weber A. N. Rieber N. Radsak M. von Bernuth H. (2015). Neutrophils: Between host defence, immune modulation, and tissue injury. PLoS Pathog. 11, 1004651. 10.1371/journal.ppat.1004651 PMC4357453 25764063

[B32] Kuhn J. Lin Y. Devreotes P. N. (2021). Using live-cell imaging and synthetic biology to probe directed migration in Dictyostelium. Front. Cell. Dev. Biol. 9, 740205. 10.3389/fcell.2021.740205 34676215 PMC8523838

[B33] Lamas I. Merlini L. Vjestica A. Vincenzetti V. Martin S. G. (2020). Optogenetics reveals Cdc42 local activation by scaffold-mediated positive feedback and Ras GTPase. PLoS Biol. 18, 3000600. 10.1371/journal.pbio.3000600 PMC7002011 31978045

[B34] Lawson C. D. Ridley A. J. (2018). Rho GTPase signaling complexes in cell migration and invasion. J. Cell. Biol. 217, 447–457. 10.1083/jcb.201612069 29233866 PMC5800797

[B35] Li X. Miao Y. Pal D. S. Devreotes P. N. (2020). Excitable networks controlling cell migration during development and disease. Semin. Cell. Dev. Biol. 100, 133–142. 10.1016/j.semcdb.2019.11.001 31836289 PMC7071959

[B36] Li X. Pal D. S. Biswas D. Iglesias P. A. Devreotes P. N. (2021). Reverse fountain flow of phosphatidylinositol-3,4-bisphosphate polarizes migrating cells. EMBO J. 40, 105094. 10.15252/embj.2020105094 PMC7883298 33586225

[B37] Luster A. D. Alon R. von Andrian U. H. (2005). Immune cell migration in inflammation: Present and future therapeutic targets. Nat. Immunol. 6, 1182–1190. 10.1038/ni1275 16369557

[B38] McFann S. Dutta S. Toettcher J. E. Shvartsman S. Y. (2021). Temporal integration of inductive cues on the way to gastrulation. Proc. Natl. Acad. Sci. U. S. A. 118, 2102691118. 10.1073/pnas.2102691118 PMC8201965 34083443

[B39] Miao Y. Bhattacharya S. Banerjee T. Abubaker-Sharif B. Long Y. Inoue T. (2019). Wave patterns organize cellular protrusions and control cortical dynamics. Mol. Syst. Biol. 15, 8585. 10.15252/msb.20188585 PMC6413885 30858181

[B40] Morgens D. W. Deans R. M. Li A. Bassik M. C. (2016). Systematic comparison of CRISPR/Cas9 and RNAi screens for essential genes. Nat. Biotechnol. 34, 634–636. 10.1038/nbt.3567 27159373 PMC4900911

[B41] Nalbant P. Chang Y. C. Birkenfeld J. Chang Z. F. Bokoch G. M. (2009). Guanine nucleotide exchange factor-H1 regulates cell migration via localized activation of RhoA at the leading edge. Mol. Biol. Cell. 20, 4070–4082. 10.1091/mbc.e09-01-0041 19625450 PMC2743625

[B42] O'Neill P. R. Castillo-Badillo J. A. Meshik X. Kalyanaraman V. Melgarejo K. Gautam N. (2018). Membrane flow drives an adhesion-independent amoeboid cell migration mode. Dev. Cell. 46, 9–22. 10.1016/j.devcel.2018.05.029 29937389 PMC6048972

[B43] O'Neill P. R. Gautam N. (2014). Subcellular optogenetic inhibition of G proteins generates signaling gradients and cell migration. Mol. Biol. Cell. 25, 2305–2314. 10.1091/mbc.E14-04-0870 24920824 PMC4116304

[B44] O'Neill P. R. Kalyanaraman V. Gautam N. (2016). Subcellular optogenetic activation of Cdc42 controls local and distal signaling to drive immune cell migration. Mol. Biol. Cell. 27, 1442–1450. 10.1091/mbc.E15-12-0832 26941336 PMC4850032

[B45] Padilla-Rodriguez M. Parker S. S. Adams D. G. Westerling T. Puleo J. I. Watson A. W. (2018). The actin cytoskeletal architecture of estrogen receptor positive breast cancer cells suppresses invasion. Nat. Commun. 9, 2980. 10.1038/s41467-018-05367-2 30061623 PMC6065369

[B46] Pakes N. K. Veltman D. M. Rivero F. Nasir J. Insall R. Williams R. S. (2012). The Rac GEF ZizB regulates development, cell motility and cytokinesis in Dictyostelium. J. Cell. Sci. 125, 2457–2465. 10.1242/jcs.100966 22366457

[B47] Pal D. S. Banerjee T. Lin Y. de Trogoff F. Borleis J. Iglesias P. A. (2023). Actuation of single downstream nodes in growth factor network steers immune cell migration. Dev. Cell. 58 (13) (Online ahead of Print). 10.1016/j.devcel.2023.04.019 PMC10524337 37220748

[B48] Pal D. S. Li X. Banerjee T. Miao Y. Devreotes P. N. (2019). The excitable signal transduction networks: Movers and shapers of eukaryotic cell migration. Int. J. Dev. Biol. 63, 407–416. 10.1387/ijdb.190265pd 31840779 PMC6956983

[B49] Pankov R. Endo Y. Even-Ram S. Araki M. Clark K. Cukierman E. (2005). A Rac switch regulates random versus directionally persistent cell migration. J. Cell. Biol. 170, 793–802. 10.1083/jcb.200503152 16129786 PMC2171343

[B50] Park K. C. Rivero F. Meili R. Lee S. Apone F. Firtel R. A. (2004). Rac regulation of chemotaxis and morphogenesis in Dictyostelium. EMBO J. 23, 4177–4189. 10.1038/sj.emboj.7600368 15470506 PMC524383

[B51] Pylayeva-Gupta Y. Grabocka E. Bar-Sagi D. (2011). RAS oncogenes: Weaving a tumorigenic web. Nat. Rev. Cancer 11, 761–774. 10.1038/nrc3106 21993244 PMC3632399

[B52] Repina N. A. Rosenbloom A. Mukherjee A. Schaffer D. V. Kane R. S. (2017). At light speed: Advances in optogenetic systems for regulating cell signaling and behavior. Annu. Rev. Chem. Biomol. Eng. 8, 13–39. 10.1146/annurev-chembioeng-060816-101254 28592174 PMC5747958

[B53] Rich A. Fehon R. G. Glotzer M. (2020). Rho1 activation recapitulates early gastrulation events in the ventral, but not dorsal, epithelium of Drosophila embryos. Elife 9, 56893. 10.7554/eLife.56893 PMC7717907 33200987

[B54] Ridley A. J. (2015). Rho GTPase signalling in cell migration. Curr. Opin. Cell. Biol. 36, 103–112. 10.1016/j.ceb.2015.08.005 26363959 PMC4728192

[B55] Ross B. Mehta S. Zhang J. (2016). Molecular tools for acute spatiotemporal manipulation of signal transduction. Curr. Opin. Chem. Biol. 34, 135–142. 10.1016/j.cbpa.2016.08.012 27639090 PMC5107343

[B56] Rossi A. Kontarakis Z. Gerri C. Nolte H. Holper S. Kruger M. (2015). Genetic compensation induced by deleterious mutations but not gene knockdowns. Nature 524, 230–233. 10.1038/nature14580 26168398

[B57] Sancak Y. Peterson T. R. Shaul Y. D. Lindquist R. A. Thoreen C. C. Bar-Peled L. (2008). The Rag GTPases bind raptor and mediate amino acid signaling to mTORC1. Science 320, 1496–1501. 10.1126/science.1157535 18497260 PMC2475333

[B58] Schneider C. A. Rasband W. S. Eliceiri K. W. (2012). NIH image to ImageJ: 25 years of image analysis. Nat. Methods 9, 671–675. 10.1038/nmeth.2089 22930834 PMC5554542

[B59] Schwechter B. Rosenmund C. Tolias K. F. (2013). RasGRF2 Rac-GEF activity couples NMDA receptor calcium flux to enhanced synaptic transmission. Proc. Natl. Acad. Sci. U. S. A. 110, 14462–14467. 10.1073/pnas.1304340110 23940355 PMC3761609

[B60] SenGupta S. Parent C. A. Bear J. E. (2021). The principles of directed cell migration. Nat. Rev. Mol. Cell. Biol. 22, 529–547. 10.1038/s41580-021-00366-6 33990789 PMC8663916

[B61] Shcherbakova D. M. Baloban M. Emelyanov A. V. Brenowitz M. Guo P. Verkhusha V. V. (2016). Bright monomeric near-infrared fluorescent proteins as tags and biosensors for multiscale imaging. Nat. Commun. 7, 12405. 10.1038/ncomms12405 27539380 PMC4992171

[B62] Stainier D. Y. Kontarakis Z. Rossi A. (2015). Making sense of anti-sense data. Dev. Cell. 32, 7–8. 10.1016/j.devcel.2014.12.012 25584794

[B63] Suire S. Lecureuil C. Anderson K. E. Damoulakis G. Niewczas I. Davidson K. (2012). GPCR activation of Ras and PI3Kc in neutrophils depends on PLCb2/b3 and the RasGEF RasGRP4. EMBO J. 31, 3118–3129. 10.1038/emboj.2012.167 22728827 PMC3400018

[B64] Taslimi A. Zoltowski B. Miranda J. G. Pathak G. P. Hughes R. M. Tucker C. L. (2016). Optimized second-generation CRY2-CIB dimerizers and photoactivatable Cre recombinase. Nat. Chem. Biol. 12, 425–430. 10.1038/nchembio.2063 27065233 PMC4871718

[B65] Toettcher J. E. Weiner O. D. Lim W. A. (2013). Using optogenetics to interrogate the dynamic control of signal transmission by the Ras/Erk module. Cell. 155, 1422–1434. 10.1016/j.cell.2013.11.004 24315106 PMC3925772

[B66] Uhlenbrock K. Eberth A. Herbrand U. Daryab N. Stege P. Meier F. (2004). The RacGEF Tiam1 inhibits migration and invasion of metastatic melanoma via a novel adhesive mechanism. J. Cell. Sci. 117, 4863–4871. 10.1242/jcs.01367 15340013

[B67] Valon L. Etoc F. Remorino A. di Pietro F. Morin X. Dahan M. (2015). Predictive spatiotemporal manipulation of signaling perturbations using optogenetics. Biophys. J. 109, 1785–1797. 10.1016/j.bpj.2015.08.042 26536256 PMC4643200

[B68] Valon L. Marin-Llaurado A. Wyatt T. Charras G. Trepat X. (2017). Optogenetic control of cellular forces and mechanotransduction. Nat. Commun. 8, 14396. 10.1038/ncomms14396 28186127 PMC5309899

[B69] Veltman D. M. Keizer-Gunnink I. Haastert P. J. (2009). An extrachromosomal, inducible expression system for *Dictyostelium discoideum* . Plasmid 61, 119–125. 10.1016/j.plasmid.2008.11.002 19046986

[B70] Wagner E. Glotzer M. (2016). Local RhoA activation induces cytokinetic furrows independent of spindle position and cell cycle stage. J. Cell. Biol. 213, 641–649. 10.1083/jcb.201603025 27298323 PMC4915195

[B71] Wang X. He L. Wu Y. I. Hahn K. M. Montell D. J. (2010). Light-mediated activation reveals a key role for Rac in collective guidance of cell movement *in vivo* . Nat. Cell. Biol. 12, 591–597. 10.1038/ncb2061 20473296 PMC2929827

[B72] Wu Y. I. Frey D. Lungu O. I. Jaehrig A. Schlichting I. Kuhlman B. (2009). A genetically encoded photoactivatable Rac controls the motility of living cells. Nature 461, 104–108. 10.1038/nature08241 19693014 PMC2766670

[B73] Wu Y. I. Wang X. He L. Montell D. Hahn K. M. (2011). Spatiotemporal control of small GTPases with light using the LOV domain. Methods Enzymol. 497, 393–407. 10.1016/B978-0-12-385075-1.00016-0 21601095 PMC3407667

[B74] Yang J. M. Bhattacharya S. West-Foyle H. Hung C. F. Wu T. C. Iglesias P. A. (2018). Integrating chemical and mechanical signals through dynamic coupling between cellular protrusions and pulsed ERK activation. Nat. Commun. 9, 4673. 10.1038/s41467-018-07150-9 30405112 PMC6220176

[B75] Yoo S. K. Deng Q. Cavnar P. J. Wu Y. I. Hahn K. M. Huttenlocher A. (2010). Differential regulation of protrusion and polarity by PI3K during neutrophil motility in live zebrafish. Dev. Cell. 18, 226–236. 10.1016/j.devcel.2009.11.015 20159593 PMC2824622

[B76] Yusa K. Rad R. Takeda J. Bradley A. (2009). Generation of transgene-free induced pluripotent mouse stem cells by the piggyBac transposon. Nat. Methods 6, 363–369. 10.1038/nmeth.1323 19337237 PMC2677165

[B77] Zhan H. Bhattacharya S. Cai H. Iglesias P. A. Huang C. H. Devreotes P. N. (2020). An excitable Ras/PI3K/ERK signaling network controls migration and oncogenic transformation in epithelial cells. Dev. Cell. 54, 608–623. 10.1016/j.devcel.2020.08.001 32877650 PMC7505206

[B78] Zhou X. X. Chung H. K. Lam A. J. Lin M. Z. (2012). Optical control of protein activity by fluorescent protein domains. Science 338, 810–814. 10.1126/science.1226854 23139335 PMC3702057

