# Supplementary figures and images for: Optogenetic modulation of guanine nucleotide exchange factors of Ras superfamily proteins directly controls cell shape and movement
Source: Front Cell Dev Biol. 2023 Jul 10;11:1195806. doi: 10.3389/fcell.2023.1195806 (PMC10363612; doi:10.3389/fcell.2023.1195806)

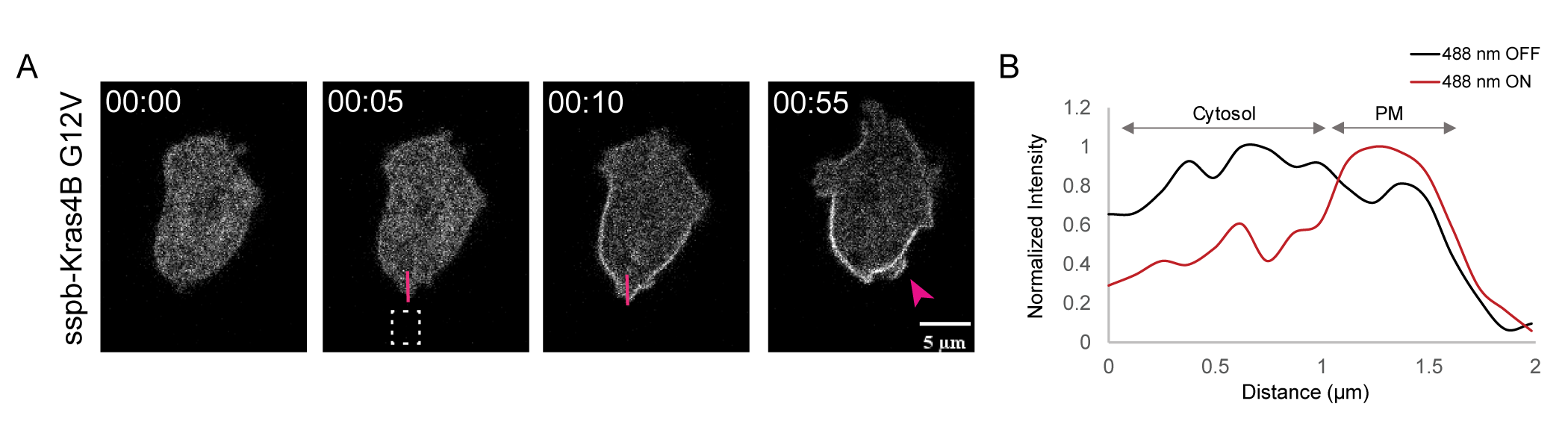

Supplement: Supplementary file 1 [file Image6.TIF]

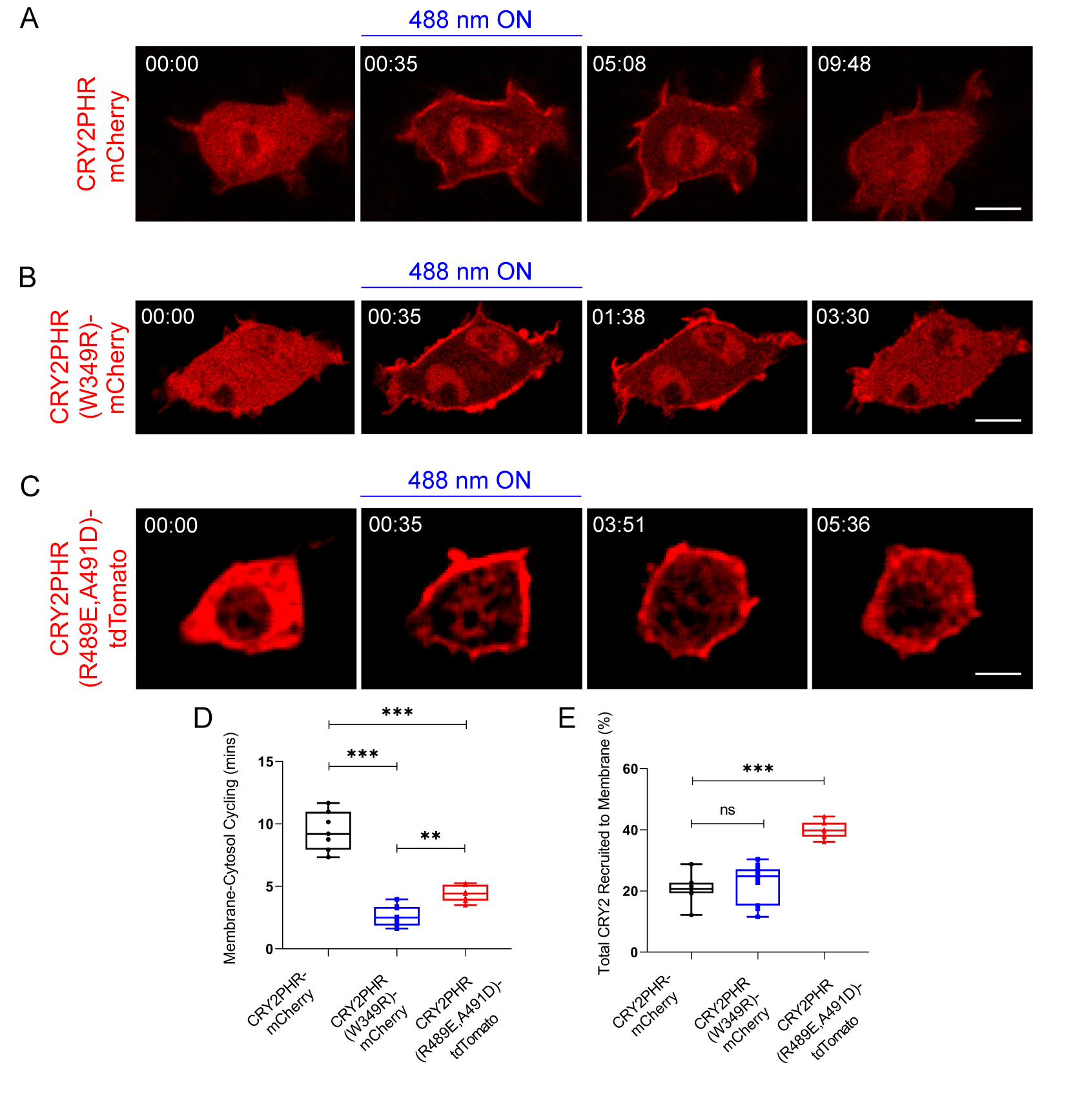

Supplement: Supplementary file 2 [file Image3.TIF]

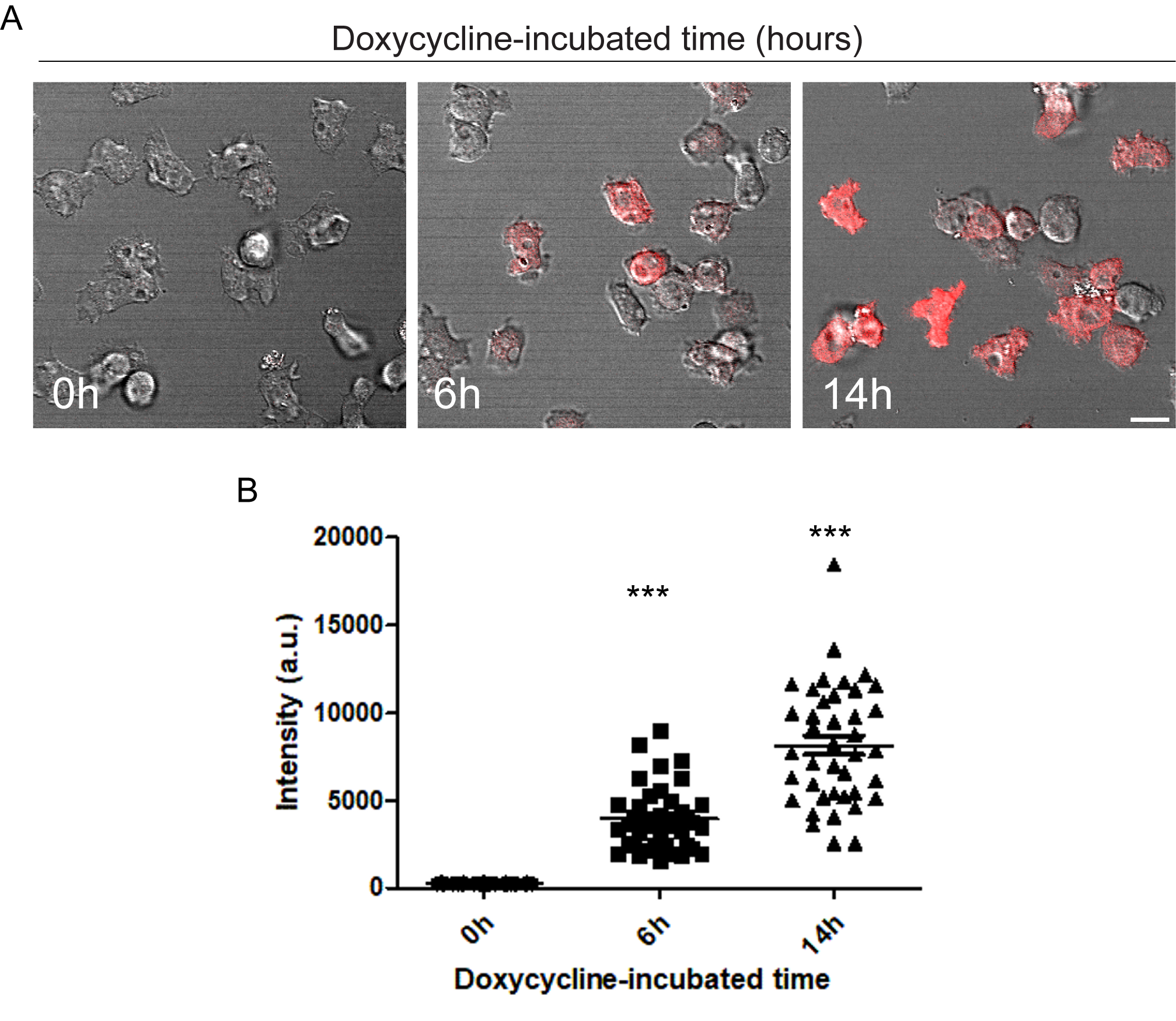

Supplement: Supplementary file 3 [file Image4.TIF]

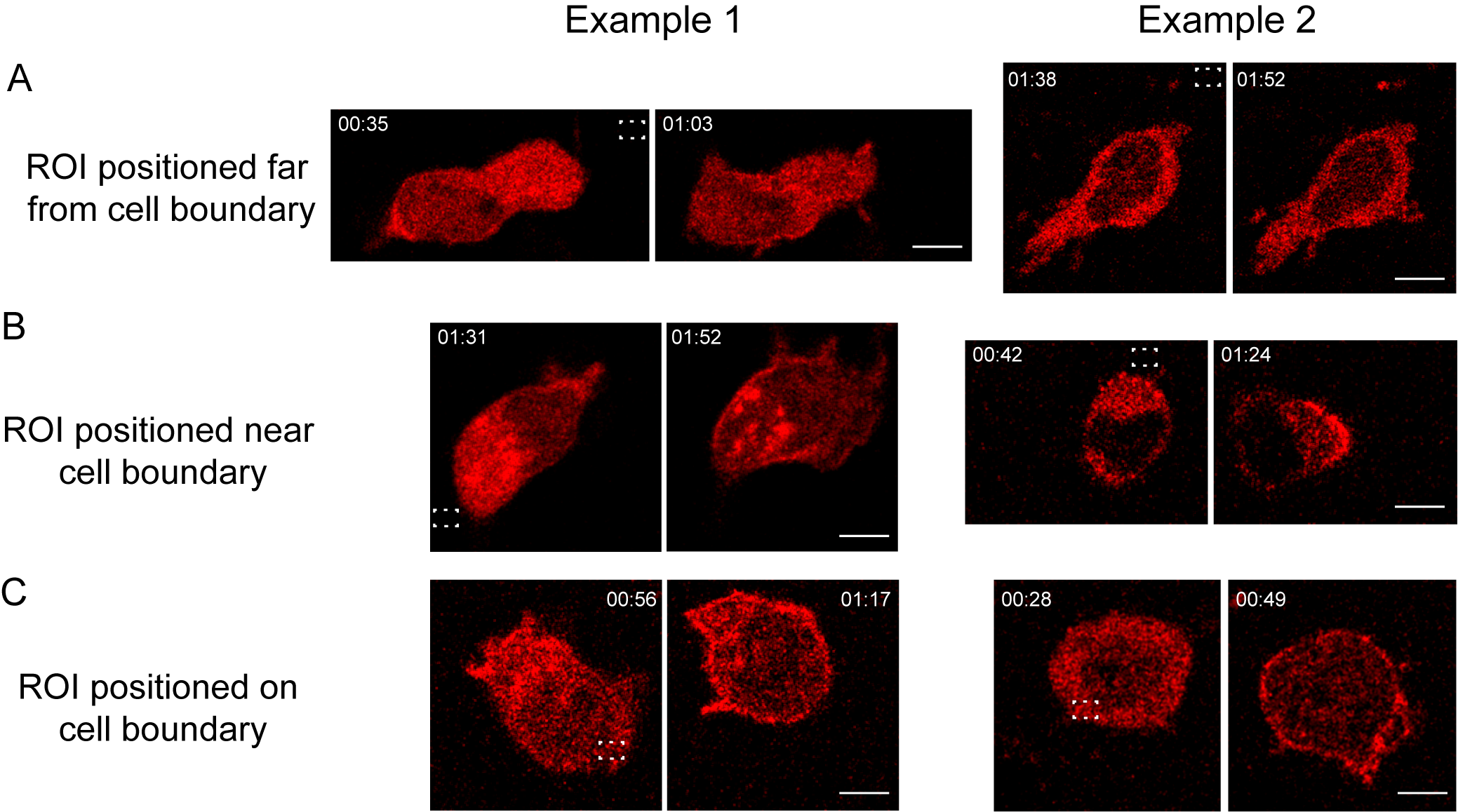

Supplement: Supplementary file 4 [file Image2.TIF]

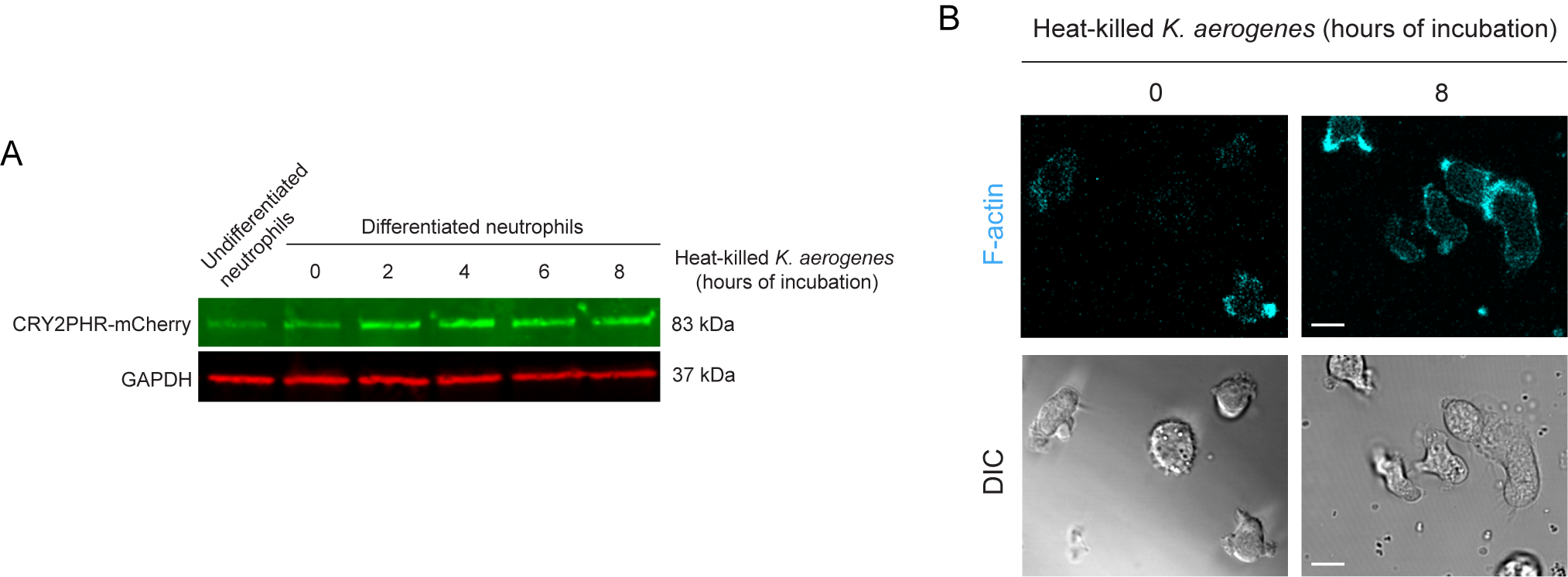

Supplement: Supplementary file 5 [file Image1.TIF]

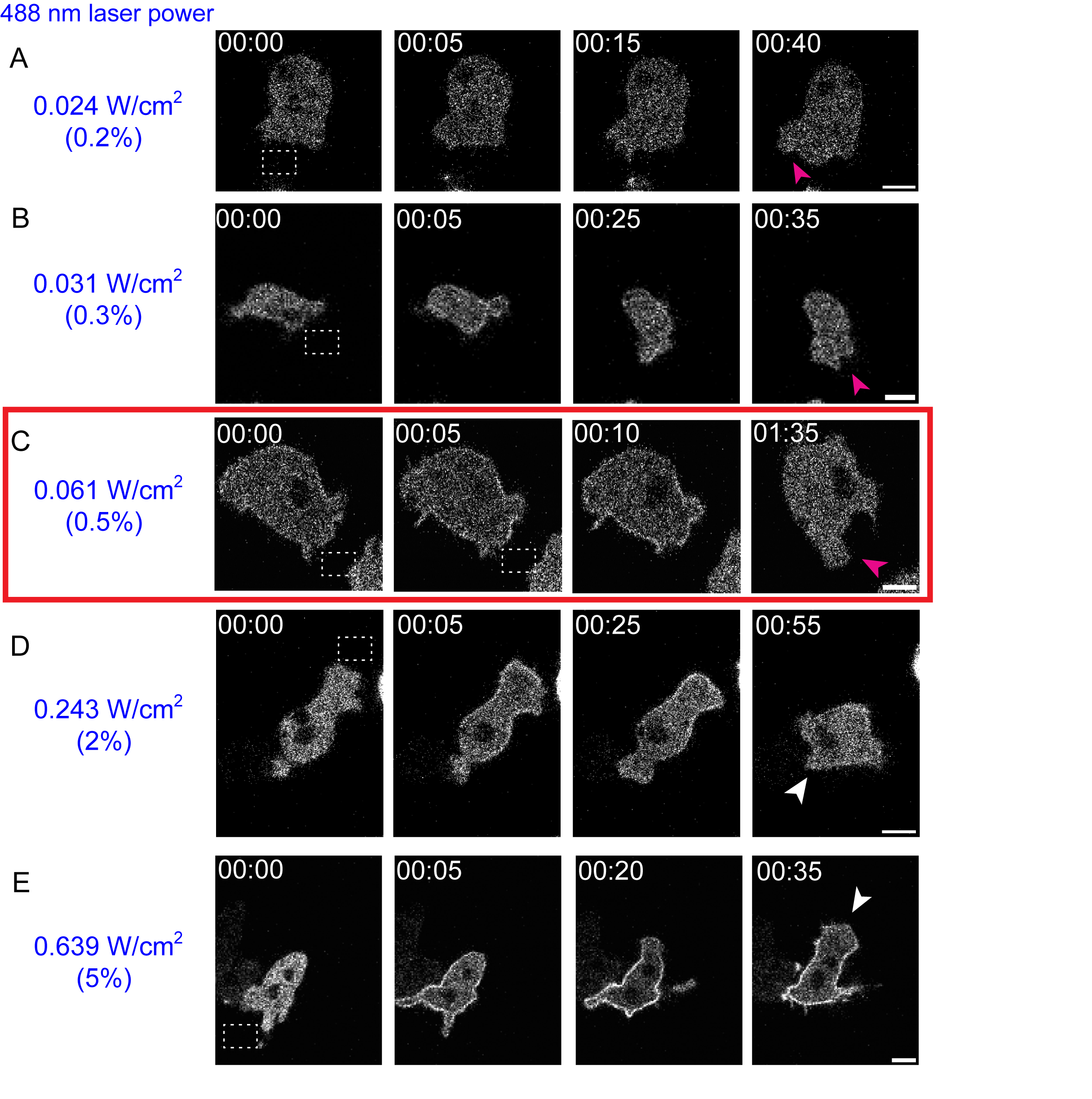

Supplement: Supplementary file 7 [file Image5.TIF]
