# Supplementary material for: Optogenetic modulation of guanine nucleotide exchange factors of Ras superfamily proteins directly controls cell shape and movement
Source: Front Cell Dev Biol. 2023 Jul 10;11:1195806. doi: 10.3389/fcell.2023.1195806 (PMC10363612; doi:10.3389/fcell.2023.1195806)
Supplement: Supplementary file 6 [file DataSheet1.PDF]

Materials and Equipment

Cell Culture Reagents

(A) HL-60 cells

Human neutrophil-like HL-60 cell line (acquired from Orion Weiner Lab at UCSF)
10 cm cell culture dish (Greiner Bio-One #664160)
Heat-killed *Klebsiella aerogenes* (DictyBase)
Heat-inactivated fetal bovine serum (FBS; Thermo Fisher Scientific #16140071)
Penicillin-streptomycin solution (Pen-Strep; Thermo Fisher Scientific #15140122)
RPMI medium 1640 (Gibco #22400-089) supplemented with 15% FBS and 1% Pen-Strep

(B) *Dictyostelium*

Dictyostelium discoideum AX2 strain (DictyBase stock).
Heat-killed *Klebsiella aerogenes* (DictyBase stock).
Doxycycline hyclate (Sigma #D9891)
10 cm cell culture dish
HL5 axenic medium: Recipe was obtained from DictyBase (http://dictybase.org). Briefly, 5g proteose peptone, 5g thiotone E peptone, 10 g glucose, 5 g yeast extract, 0.35 g $Na_2HPO_4*7H_2O$, 0.35 g KH_2PO_4, and 0.05 g dihydrostreptomycin-sulfate (GoldBio, #S-150-100) were added to distilled water and the volume was brought to 1 liter. pH was adjusted with HCl to pH 6.4-6.7. Finally, medium was autoclaved for 20 mins to sterilize.
Development buffer (DB): Recipe was obtained from DictyBase. Briefly, 5 mM Na_2HPO_4, 5 mM KH_2PO_4, 1 mM $CaCl_2$, and 2 mM $MgCl_2$ were added to distilled water. pH was adjusted to 6.5 and filter sterilized.
H-50 buffer: Recipe was obtained from DictyBase. Briefly, 20 mM HEPES, 1 mM $MgSO_4$, 50 mM KCl, 5 mM $NaHCO_3$, 10 mM NaCl, and 1 mM NaH_2PO_4 were added to distilled water. pH was adjusted to 7 and filter sterilized.

(C) MCF-10CA1h cells

MCF-10CA1h cell line (purchased from the Animal Model and Therapeutic Evaluation Core, Wayne State University)
10 cm cell culture dish
0.05% Trypsin-EDTA solution (Gibco #25300054)
DMEM/F-12 (Gibco #10565042) supplemented with 5% horse serum (Gibco #26050088), 20 ng/ml EGF (Sigma #E9644), 100 ng/ml cholera toxin (Sigma #C-8052), 0.5 mg/ml hydrocortisone (Sigma #H-0888), and 10 μg/ml insulin (Sigma #I-1882)

(D) RAW264.7 cells

RAW 264.7 mouse monocyte cells (gifted by N. Gautam Lab at WUSTL)
Cell Scraper (Greiner Bio-One; #541070)
10 cm cell culture dish
DMEM (Sigma-Aldrich; #D6429) supplemented with 10% FBS and 1% penicillin-streptomycin

(E) HEK293T cells

HEK293T Human embryonic kidney cell line (purchased from ATCC)
DMEM (Gibco #10569-010) supplemented with 10% FBS

***Dictyostelium* Development/ Neutrophil Differentiation System and Reagents**

MINIPULS 3 peristaltic pump (Gilson)
Innova 2000 platform shaker (New Brunswick)
50 ml narrow-mouth Erlenmeyer flask (Pyrex #4980-50)
Precision pump tubing, peroxide-cured silicone, L/S 25 (Avantor #MFLX96400-25)
cyclic AMP (cAMP; Sigma #A6885)
Development buffer (DB)
DMSO solution (Sigma #D2650)
10 cm cell culture dish

Immunoblotting System

Pre-cast 4-15% polyacrylamide gels (BIO-RAD #5671085)
PVDF membranes (BIO-RAD #162-0262)
Trans-Blot Turbo semi-dry transfer apparatus (BIO-RAD #1704150EDU)
Trans-Blot Turbo Transfer buffer (BIO-RAD #10026938)
Blocking buffer: Intercept buffer (Li-Cor #927-60001) mixed with 1x TBS containing 0.1% Tween 20 in equal volumes
Anti-mcherry polyclonal antibody (Invitrogen #PA5-34974)
Anti-GAPDH polyclonal antibody (Invitrogen #PA1-987)
Goat anti-rabbit IRDye 680RD-cojugated secondary antibody (Li-Cor #925-68071)
Odyssey CLx imaging system (Li-Cor)

Methods

Cell Culture

Human neutrophil-like HL-60 cell line was cultured in RPMI medium 1640 supplemented with 15% heat-inactivated FBS and 1% pen-strep. Cells were subcultured at a seeding density of 0.15 million cells/ml every 3 days[1]. Stable cell lines were grown similarly with selection antibiotics.

MCF-10CA1h cells were grown in supplemented DMEM/F-12 medium in 10 cm culture dishes. Once they reached 80% confluency, cells were subcultured, post-trypsinization with 0.05% trypsin, at a ratio of 1:10[2].

RAW 264.7 mouse monocytes were grown in supplemented DMEM medium. At ~90% confluency, cells were harvested by scraping and passaged at a ratio of 1:4[3].

HEK293T Human embryonic kidney cells were cultured in supplemented DMEM medium. Cells were grown according to ATCC guidelines.

All mammalian cells were grown at 37°C in 5% CO_2.

Wild type *Dictyostelium* (AX2 strain) were cultured at 22°C in HL5 medium supplemented with 300 μg/mL streptomycin. Cells were passaged either in suspension or on cell culture dishes every 3-4 days, and experiments were performed within 8-9 weeks of thawing cells from frozen stocks[4,5]. Stable cell lines were grown similarly with selection antibiotics.

Development and differentiation of *Dictyostelium* and neutrophils

For development, vegetative *Dictyostelium* cells were grown to a density of less than 5×10^6 cells/ml. 1×10^8 cells were harvested, washed twice in 50 ml DB, and resuspended at a concentration of 2×10^7 cells/ml in 5 ml sterile DB. Cell suspension was rotated in a 50 ml narrow-mouth flask for 1 hour at 100 rpm in RT. Using a peristaltic pump connected with appropriate tubing, cells were pulsed with 50 nM cAMP every 6 min for the next 5 hours. This allowed cells to become aggregation-competent and could then be used for migration studies[6]. Stable cell lines were differentiated similarly.

To differentiate HL-60 cells, 1.3% DMSO was introduced in a fresh cell population seeded at a density of 1.5×10^5 cells/ml. Cells along with DMSO were incubated for 5-8 days, before experimentation. HL-60 is a myeloid leukemia cell line and an effective model to investigate human neutrophils, upon differentiation[1,7]. Stable cell lines were differentiated similarly in absence of selection antibiotics.

SDS-PAGE and Western blotting

Protein samples equivalent to at least 5×10^5 cells were loaded in each well of pre-cast polyacrylamide gels (4-15%) at 120 V for 1 hour. Next, gels were transferred onto PVDF membranes for 10 mins using a semi-dry transfer apparatus. Post-transfer, membranes were incubated in blocking solution for 1 hour. CRY2PHR-mcherry2 expression (~83 kDa) was detected by incubating membrane with rabbit anti-mcherry polyclonal antibody (1:1000 in blocking solution) overnight at 4^0C. GAPDH (~37 kDA), detected by anti-GAPDH polyclonal antibody (1:1000 in blocking solution), was used as loading control. After incubation, membranes were washed five times in TBST for 5 mins each. Next, membranes were probed with goat anti-rabbit IRDye 680RD-cojugated secondary antibody (1:10,000 dilution in blocking solution) for 1 hour in the dark. Membranes were again washed five times in TBST for 5 mins each. Near-infrared signal from the membranes was visualized on the Odyssey CLx imaging system.

References

1. Millius, A., and Weiner, O.D. (2010). Manipulation of neutrophil-like HL-60 cells for the study of directed cell migration. Methods Mol Biol *591*, 147-158. 10.1007/978-1-60761-404-3_9.
2. Zhan, H., Bhattacharya, S., Cai, H., Iglesias, P.A., Huang, C.H., and Devreotes, P.N. (2020). An Excitable Ras/PI3K/ERK Signaling Network Controls Migration and Oncogenic Transformation in Epithelial Cells. Dev Cell *54*, 608-623 e605. 10.1016/j.devcel.2020.08.001.
3. Meshik, X., O'Neill, P.R., and Gautam, N. (2018). Optogenetic Control of Cell Migration. Methods Mol Biol *1749*, 313-324. 10.1007/978-1-4939-7701-7_22.
4. Basu, S., Fey, P., Pandit, Y., Dodson, R., Kibbe, W.A., and Chisholm, R.L. (2013). DictyBase 2013: integrating multiple Dictyostelid species. Nucleic Acids Res *41*, D676-683. 10.1093/nar/gks1064.
5. Li, X., Pal, D.S., Biswas, D., Iglesias, P.A., and Devreotes, P.N. (2021). Reverse fountain flow of phosphatidylinositol-3,4-bisphosphate polarizes migrating cells. EMBO J *40*, e105094. 10.15252/embj.2020105094.
6. Artemenko, Y., Swaney, K.F., and Devreotes, P.N. (2011). Assessment of development and chemotaxis in Dictyostelium discoideum mutants. Methods Mol Biol *769*, 287-309. 10.1007/978-1-61779-207-6_20.
7. Rincon, E., Rocha-Gregg, B.L., and Collins, S.R. (2018). A map of gene expression in neutrophil-like cell lines. BMC Genomics *19*, 573. 10.1186/s12864-018-4957-6.
